# Saving time maintaining reliability: a new method for quantification of *Tetranychus urticae* damage in Arabidopsis whole rosettes

**DOI:** 10.1186/s12870-020-02584-0

**Published:** 2020-08-27

**Authors:** Dairon Ojeda-Martinez, Manuel Martinez, Isabel Diaz, M. Estrella Santamaria

**Affiliations:** 1 Centro de Biotecnología y Genómica de Plantas, Universidad Politécnica de Madrid – Instituto Nacional de Investigación y Tecnología Agraria y Alimentaria, Madrid, Spain; 2 Departamento de Biotecnología-Biología Vegetal, Escuela Técnica Superior de Ingeniería Agronómica, Alimentaria y de Biosistemas, Universidad Politécnica de Madrid, Madrid, Spain

**Keywords:** *Arabidopsis thaliana*, Assess, Chlorotic spots, CompuEye, Ilastik, Fiji, Photoshop, Plant damage quantification, *Tetranychus urticae*, Machine learning

## Abstract

**Background:**

The model species *Tetranychus urticae* produces important plant injury and economic losses in the field. The current accepted method for the quantification of the spider mite damage in Arabidopsis whole rosettes is time consuming and entails a bottleneck for large-scale studies such as mutant screening or quantitative genetic analyses. Here, we describe an improved version of the existing method by designing an automatic protocol. The accuracy, precision, reproducibility and concordance of the new enhanced approach are validated in two Arabidopsis accessions with opposite damage phenotypes. Results are compared to the currently available manual method.

**Results:**

Image acquisition experiments revealed that the automatic settings plus 10 values of brightness and the black background are the optimal conditions for a specific recognition of spider mite damage by software programs. Among the different tested methods, the Ilastik-Fiji tandem based on machine learning was the best procedure able to quantify the damage maintaining the differential range of damage between accessions. In addition, the Ilastik-Fiji tandem method showed the lowest variability within a set of conditions and the highest stability under different lighting or background surroundings. Bland-Altman concordance results pointed out a negative value for Ilastik-Fiji, which implies a minor estimation of the damage when compared to the manual standard method.

**Conclusions:**

The novel approach using Ilastik and Fiji programs entails a great improvement for the quantification of the specific spider mite damage in Arabidopsis whole rosettes. The automation of the proposed method based on interactive machine learning eliminates the subjectivity and inter-rater-variability of the previous manual protocol. Besides, this method offers a robust tool for time saving and to avoid the damage overestimation observed with other methods.

## Background

The two spotted spider mite *Tetranychus urticae* Koch (Acari*:* Tetranychidae) is a cosmopolitan phytophagous pest that causes important plant damages and yield losses [1]. The predicted expansion of the spider mites under the climate change, its extreme polyphagous character with more than 1100 documented host plants and its ability to develop pesticide resistance makes *T. urticae* one of the most significant pests in the agriculture [1–3]. Phytophagous mites pierce parenchymatic plant cells using stylets to suck their nutrients and produce severe chlorosis mainly on the leaves leading to a reduction in crop yield [4–6]. *T. urticae* is a model within chelicerate herbivores with its small genome sequenced and a broad range of tools and protocols developed [1, 7–9]. Besides, the mite ability to feed on the model species *Arabidopsis thaliana* has provided an outstanding opportunity for functional studies about plant-mite interactions [10–16].

The quantification of the plant damage produced by *T. urticae* is particularly important to decision-makers when the crop damage is related to yield losses; for plant breeding approaches where various accessions, germplasm, varieties and/or cultivars need to be rated; and for pest management decisions [17]. Likewise, it is a valuable tool for the understanding of fundamental processes in biology, such as plant-pest coevolution [18]. During last years, the quantification of the damage produced by *T. urticae* in Arabidopsis plants [10–16] has been measured using the method described by Cazaux et al. [7]. This method is based on the manual identification of the chlorotic spots by using the Adobe Photoshop program and the later transformation of the pixels in mm^2^ of damaged area. This manual approach is very subjective because the rater (human specialist) has to distinguish the chlorotic spots produced by the mite feeding from other light coloured or background areas such as trichomes or early senescence symptoms. Even when this manual method provides an accurate and precise quantification, it is time-consuming and entails the intrinsic intra- and inter-rater variability [19]. In the case of the symptoms caused by pathogens or chewing insects, the high contrast between damaged/undamaged regions facilitates the repeatability and reproducibility of the results using automatic programs such as Fiji [13, 20] or APS Assess [21, 22]. In contrast, cell content feeders (mites or thrips) and phloem feeders (aphids) produce subtle symptoms difficult to recognise by automatic software programs. In previous works, the software CompuEye [23], Ilastik [24] and/or Fiji [25] have been used to quantify the cell damage produced by sucking feeders. These approaches have been used to evaluate plant damage in detached leaves or in a piece of leaf of known area, but not in whole plants which should provide a more realistic measure. Therefore, we decided to test the effectiveness of the three automatic programs (Assess, CompuEye and the tandem Ilastik-Fiji) in parallel to the manual annotation (Photoshop) to estimate the damage caused by spider mites on Arabidopsis rosettes. These three automatic programs were selected based on the availability of the software, their user-friendly platforms and their capacity to process in batch mode large amounts of images. The software selection covers well documented methods with the sensitivity to identify subtle biological stresses, employing strategies to do so such as simple thresholding (Assess and CompuEye) and modern machine learning techniques (Ilastik). Another important considered feature for the selection was the accessibility of the methodologies to non-computer scientists, so they could be used without resorting to programming expertise or high computer processing power. Additionally, the effects of the background and the selected lighting conditions during the image acquisition have been analysed to optimise this process. Our comparative analysis highlighted advantages and limitations of each approach compared to the manual method and demonstrated that the tandem Ilastik-Fiji method was the most reliable. The automation of the procedure, by using modern machine learning methodologies, eliminated the intra and inter-rater variability, massively reduced the quantification process time, and avoided the overestimation of the damage inherent to the manual method.

## Results

### Selection and optimization of methods under study

Three automatic software programs were selected for this study, Assess [22], CompuEye [23] and the tandem Ilastik-Fiji [24]. These programs were identified as previously used for damage quantification with potential to automatically discriminate the subtle damage produced by the *T. urticae* infestation. As a reference, we used the manual annotation [7] currently used to quantify spider mite damage in Arabidopsis. For the analysis on Assess 2.0, the Classic Panel was used. The thresholds on each colour plane were explored to discriminate the rosette area from the background. Damage analysis was performed on the rosette area by also exploring the threshold values on each plane. Once the desired plane and threshold values were identified (Additional file 1: Table S1), macroinstructions (macros) were designed to automatically process the images (Additional file 2: Macro S1). CompuEye analysis was performed by testing each of the four available systems. Different degrees of sensitivity were assayed to correctly identify the damaged areas. Once the combination of system/sensitivity was identified (Additional file 1: Table S2), the bulk of infested and non-infested plant images was analysed. The custom detection system of this program was not used due to poor results.

**Fig. 1:**
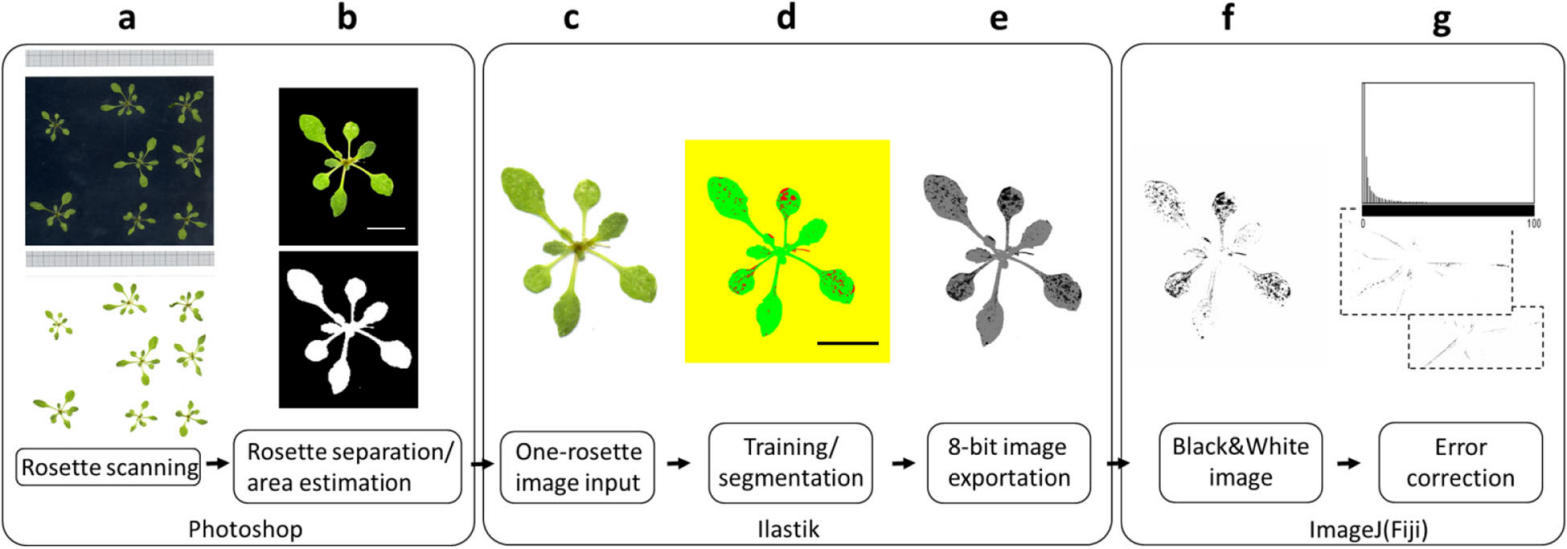
Flow chart of the analysis procedure using the Ilastik-Fiji method. Eight rosettes for control or treated rosettes were scanned either on black or white background (**a**), rosettes are individualized, and the total area of each rosette is estimated using Photoshop (**b**). A selection of individual rosettes is imported to Ilastik (**c**) and used to train the program (**d**), to distinguish mite damage (red), from healthy rosette (green) and background (yellow). All the images are processed and exported as 8-bit (**e**). The previous images are imported into Fiji and the damaged areas extracted and exported as black and white images (**f**). The damaged area is calculated for treated and control rosettes (**g**). Control rosettes are then used to correct the damage area from mite-treated plants. Black and white scale bars indicate 1 cm

Regarding Ilastik, the program was trained to identify the damaged tissue, and the original images were segmented using this information. The segmented images were processed in Fiji (Additional file 2: Macros S2-S4). Control rosette images were used to select the pixel cluster size threshold to discriminate background noise. A cluster size of 37 pixels was detected as the mean value under which damage was disregarded for the final measurements (Additional file 2: Fig. S1). A workflow of the entire analysis procedure is showed in Fig. 1 and a detailed explanation in the methods section.

The identification of specific thresholds for each lighting and background condition was necessary for all the automatic methods. Furthermore, control rosettes were used to correct for the average error that the automatic methods committed. This error consists on the identification of “damaged” tissue in control rosettes due to the presence of confounding areas related to the light colour associated to young leaves, trichomes or early senescence.

### Scanning condition optimization

**Fig. 2:**
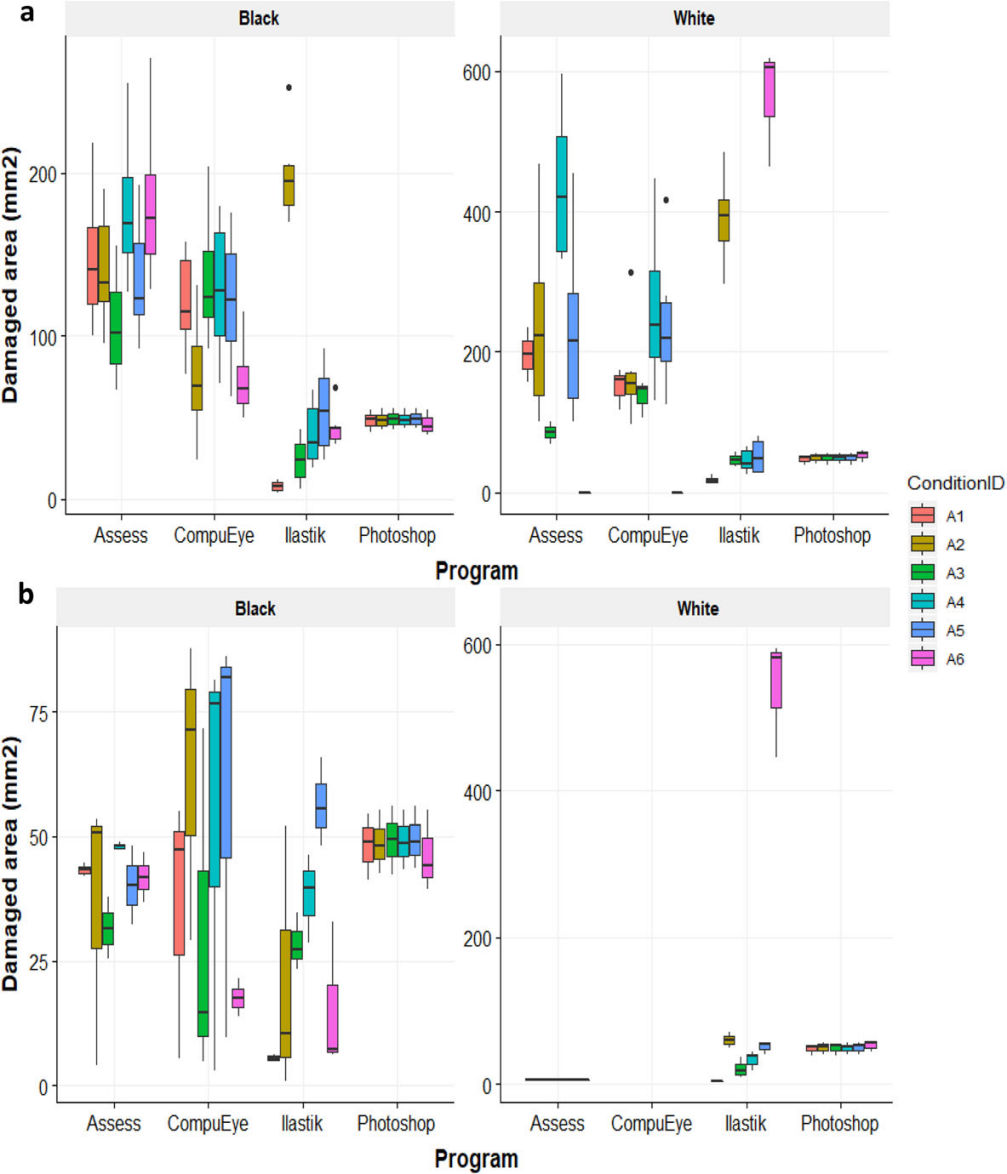
Box-and-whiskers plots representing the estimated damaged area (mm^2^). Data were obtained from each system under different lighting conditions and white and black backgrounds: (**a**) Without control rosette correction, and (**b**) after error correction using rosette controls. Data were obtained from *A. thaliana* Col-0 genotype, infested with 50 *T. urticae* adults for 4 days; *n* = 3. Lighting conditions (Brightness, Contrast for each case): A1 = 1,-56; A2 = 50,-25; A6 = 90,-100; A4 = Automatic threshold (30,-69 White; 40,-69 Black); A3 and A5 values were selected for each background subtracting and adding 10 values of brightness, respectively, maintaining contrast values. Black dots indicate outlier values

To analyse the effect of lighting variations on the ability of the methods used to identify damage, six brightness/contrast combinations and two backgrounds were applied to each rosette image (Fig. 2). Attention was paid to the data closeness of the automatic methods to the manual reference method and the variability within each method and condition tested (Fig. 2).

Before the values were corrected using control rosettes (Fig. 2a), a clear damage overestimation and variability were detected on the values calculated by Assess and CompuEye for all of the lighting conditions and backgrounds tested. The damage was three/four times higher than the calculated with the reference method for the black background and up to nine-fold when the photos were scanned with white background. Values obtained with the Ilastik-Fiji tandem were closer to the reference programme results showing an overestimation only in the condition A2 with black background and in A2 and A6 with white background. When data were corrected using the damage of the control rosettes (Fig. 2b), on the black background Assess and CompuEye overestimation decreased and maintained high variability for most of the conditions tested. Damage estimated by the Ilastik-Fiji tandem was again closer to the reference method, and showed an underestimation for the conditions A1, A2 and A6. When the correction was applied to the images taken under the white background, the damage quantified with Assess and CompuEye showed a significant reduction or an absence of values due to the identification of more damage in the control than in the infested rosettes. Regarding the Ilastik-Fiji tandem, the damage was overestimated only in the condition A6, being the values closer to the ones calculated with the reference program, and with an acceptable intra and inter variability (Additional file 1: Table S3). Statistical analysis using GLM revealed significant variations on the damage identified by the programs for background (χ^2^ = 29.99, *p* < 0.001) and lighting condition (χ^2^ = 63.85, p < 0.001). No difference was found for the damage estimated under the same lighting conditions on different backgrounds (χ^2^ = 5.14, *p* = 0.27) (Additional file 3: Table S1). The program with the closest values to the Photoshop reference method was the Ilastik-Fiji tandem, in particular for the conditions A3, A4 and A5, which also had low Standard Deviations (SDs) and Coefficients of Variation (CVs), and behaved similarly for both backgrounds (Additional file 1: Table S3). Under these same three conditions, Assess had also damage values on the black background with low SDs and CVs that were close to the Photoshop standard. Any lighting condition out of A3, A4 and A5 induced the automatic methods to either overestimate or underestimate the damage values, and also produced high variability or excessive damage identification on the control rosettes. Due to the accuracy of the results of two out of the three automatic procedures, A3, A4 and A5 conditions were selected for subsequent analyses.

### Accuracy and precision of each method

**Fig. 3:**
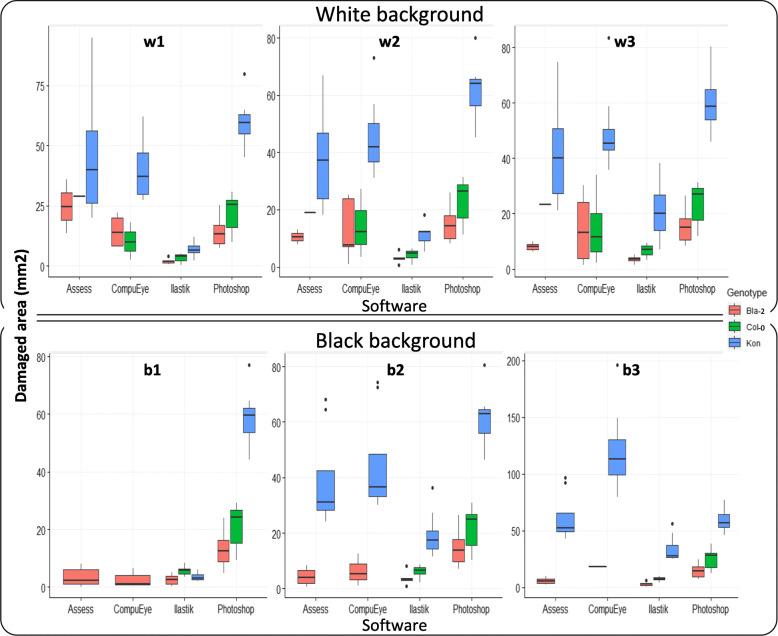
Box-and-whiskers plots representing the estimated damaged area (mm^2^) obtained from each of the systems under different lighting conditions and white (top row) and black (lower row) backgrounds. Data were obtained from *A. thaliana* Col-0, Bla-2 and Kon genotypes, infested with 20 *T. urticae* adults for 4 days; *n* = 8. Lighting conditions (brightness, contrast for each case): **w2** and **b2** (central column) = Automatic threshold (30,-20 White; 40,-10 Black); **w1**, **w3**, **b1** and **b3** values were selected for each background subtracting and adding 10 values of brightness, respectively, maintaining contrast values. Black dots indicate outlier values

In order to test the precision and accuracy of each method, two additional genotypes, Kon and Bla-2, were added to the Col-0 used in the previous experiment. Kon plants were highly susceptible to the attack of the spider mite, displaying more chlorotic areas, while Bla-2 was highly resistant showing less symptoms on the infested rosette. The response of Col-0 and Bla-2 rosettes to the spider mite was quite similar, although Col-0 was a bit less resistant [11]. Accuracy was assessed by analysing the position of the genotypes in the plant damage scale and calculating the quotient between the Kon/Col-0 mean damage [13, 14]. For every lighting condition tested on both backgrounds, the mean damage calculated by all the methods was below the standard, except for the Kon genotype on the condition b3 (Fig. 3). The worst accuracy scenario occurred for the least lighted b1 condition, for which Assess and CompuEye lost the information because they detected more “damaged” areas on the control than on infested rosettes (Fig. 3). The CompuEye and Assess outcomes under conditions b2 and b3 were also misleading because of some loss of information. In the conditions w2 and w3 the CompuEye results were the expected comparing the three accessions (Kon > Col-0 ~ Bla-2) but the SDs and CVs were quite high (Fig. 3; Additional file 1: Table S4). Regarding Ilastik, the damaged areas displayed in five out of the six conditions maintained the standard known relationship among the mean damage for each genotype (Kon > Col-0 ~ Bla-2). However, their values were always lower than in the manual annotation (Fig. 3; Additional file 1: Table S4; Additional file 3: Table S2). The condition where the Ilastik results reproduced better the known relationship among the genotypes was b3 on the black background. In the aforementioned condition, the relationship of the susceptible genotype (Kon) divided by the resistant one (Col-0) reached 4.5 (Table 1), which fitted with previous manual-obtained data where the quotient Kon/Col-0 was between 2.5 and 4.5 [13, 14].

**Table 1:** Kon/Col-0 mean damage quotient calculated for each automatic method and the standard (Photoshop)

Program	Assess	CompuEye	Ilastik	Photoshop
Lighting Condition				
W1	–	3.9	2.0	2.7
W2	–	3.2	2.7	2.7
W3	–	3.4	3.1	2.5
B1	–	–	0.6	2.8
B2	–	–	3.1	2.8
B3	–	–	4.5	2.3

The values represent the relationship between the two genotypes, Kon and Bla-2, on the opposite ends of the spider mite resistance spectrum.

In addition, the Ilastik method had less data variability, being its SDs and CVs lower compared to the other automatic methods (Fig. 3; Additional file 1: Table S4). All the methods displayed more dispersed values for Kon than for the other two genotypes (Fig. 3; Additional file 1: Table S4).

### Background and lighting effects on the method reproducibility

**Fig. 4:**
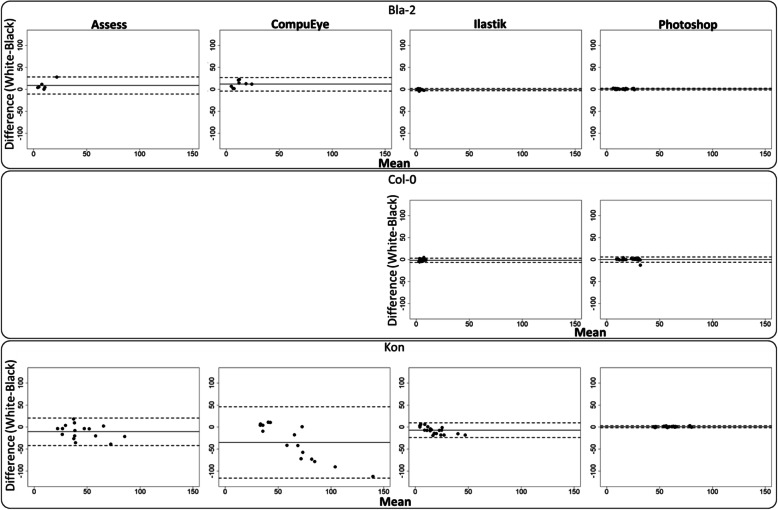
Bland-Altman plot comparing reproducibility of the three automatic methods and the standard on different backgrounds. Each panel compares the results of the methods on white versus black background for the genotypes Bla-2 (upper panel), Col-0 (middle panel) and Kon (lower panel). Method identification is on top of its corresponding column. The Y axis represents the damage values on white background minus the values estimated on the black background. The central solid line indicates the mean difference, the two outer dashed lines indicate ±1.96 standard deviations

To evaluate the reproducibility of the automatic methods, damage estimation was conducted using three lighting conditions on two backgrounds. A GLM was performed to detect statistical differences and a pair-wise comparison analysis with Bonferroni correction to locate them. B&A plots were produced for each method to compare the estimated variability between backgrounds (Fig. 4). The coefficient of repeatability was also calculated to analyse the effect of background and lighting conditions (Tables 2 and 3). As expected, statistical differences were detected for genotypes (χ^2^ = 72.11, *p* < 0.001), lighting conditions (χ^2^ = 21.8, p < 0.001) and programs (χ^2^ = 76.01, p < 0.001) (Additional file 3: Table S3). Lighting conditions varied the damage estimated by the programs (χ^2^ = 42.95, p < 0.001). Differences were also detected among the programs for the damage calculated in the genotypes (χ^2^ = 29.22, p < 0.001).

**Table 2:** Coefficients of repeatability (CR) and confidence intervals (CI) when lighting conditions are changed

Program		Assess		CompuEye		Ilastik		Photoshop	
Conditions									
Genotype	Background	CR	CI	CR	CI	CR	CI	CR	CI
Bla-2	White	20.38	13.23;48.37	6.88	4.94;11.77	2.10	1.59;3.19	1.31	1.02;1.85
Col-0		9.98	5.77;44.10	9.87	6.81;18.92	3.69	2.88;5.23	1.57	1.22;2.22
Kon		20.83	16.25;29.53	11.71	9.13;16.61	16.55	12.91;23.47	7.43	5.80;10.54
Bla-2	Black	1.15	0.59;2.22	12.90	7.45;56.96	1.77	1.36;2.59	2.60	2.03;3.69
Col-0		–	–	–	–	2.26	1.76;3.21	6.80	5.30;9.64
Kon		32.16	25.09;45.59	110.51	86.20;156.66	31.56	23.32;50.28	7.57	5.91;10.74

**Table 3:** Coefficients of repeatability (CR) and confidence intervals (CI) when background is changed

Program	Assess		CompuEye		Ilastik		Photoshop	
	CR	CI	CR	CI	CR	CI	CR	CI
Genotype								
Bla-2	17.70	12.21;33.90	19.14	13.74;32.74	1.84	1.47;2.50	1.76	1.43;2.32
Col-0	–	–	–	–	4.13	3.36;5.44	4.20	3.41;5.54
Kon	26.07	20.33;36.96	73.54	57.36;104.25	14.99	12.02;20.18	1.94	1.57;2.56

According to B&A plots and Coefficient of Repeatability (CR) values, the Ilastik estimations were more reproducible with different backgrounds. Ilastik displayed narrower limits of agreement compared to the other two automatic methods for every genotype analysed (Fig. 4). CR values for different lighting conditions (Table 2) and background changes (Table 3) were in general lower for Ilastik compared to the other two automatic methods, especially for Bla-2 and Col-0 genotypes.

**Fig. 5:**
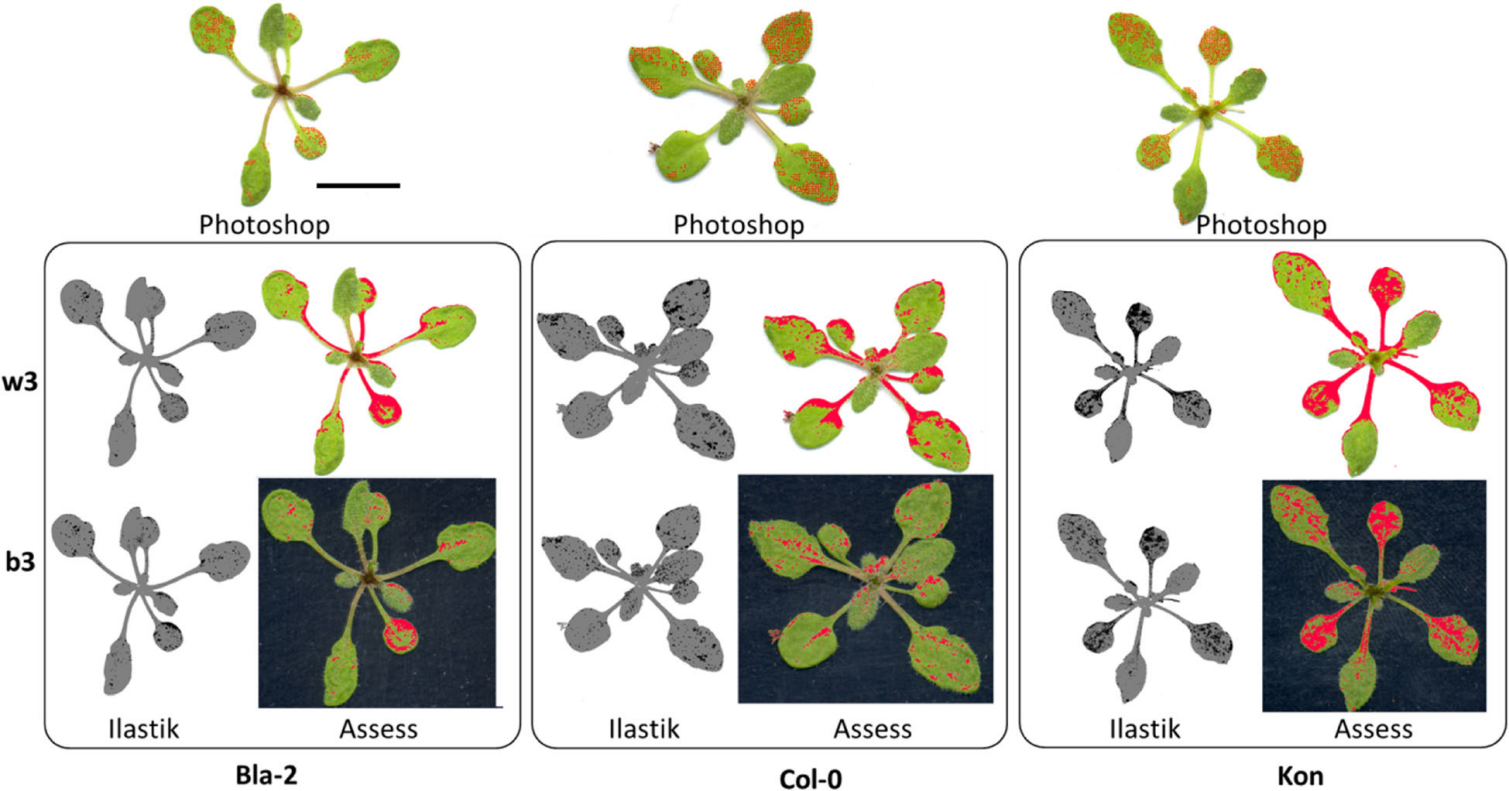
Area selection accuracy on two lighting conditions and backgrounds. The identification of damaged areas by the two software’s that has the best accuracy (Ilastik and Assess), is assessed using the standard (Photoshop, top row). Results are shown for the brightest conditions on both backgrounds: w3 (white background, middle row) and b3 (black background, lower row). Damaged areas are represented by red colour (Assess and Photoshop) and black colour (Ilastik). Lighting conditions (brightness, contrast for each case): w3 and b3 (40,-20 White; 50,-10 Black, respectively). Black scale bar indicates 1 cm

In addition to all the previous data, the damage quantification images generated by Ilastik had the highest visual accuracy (Fig. 5).

### Concordance analysis

**Fig. 6:**
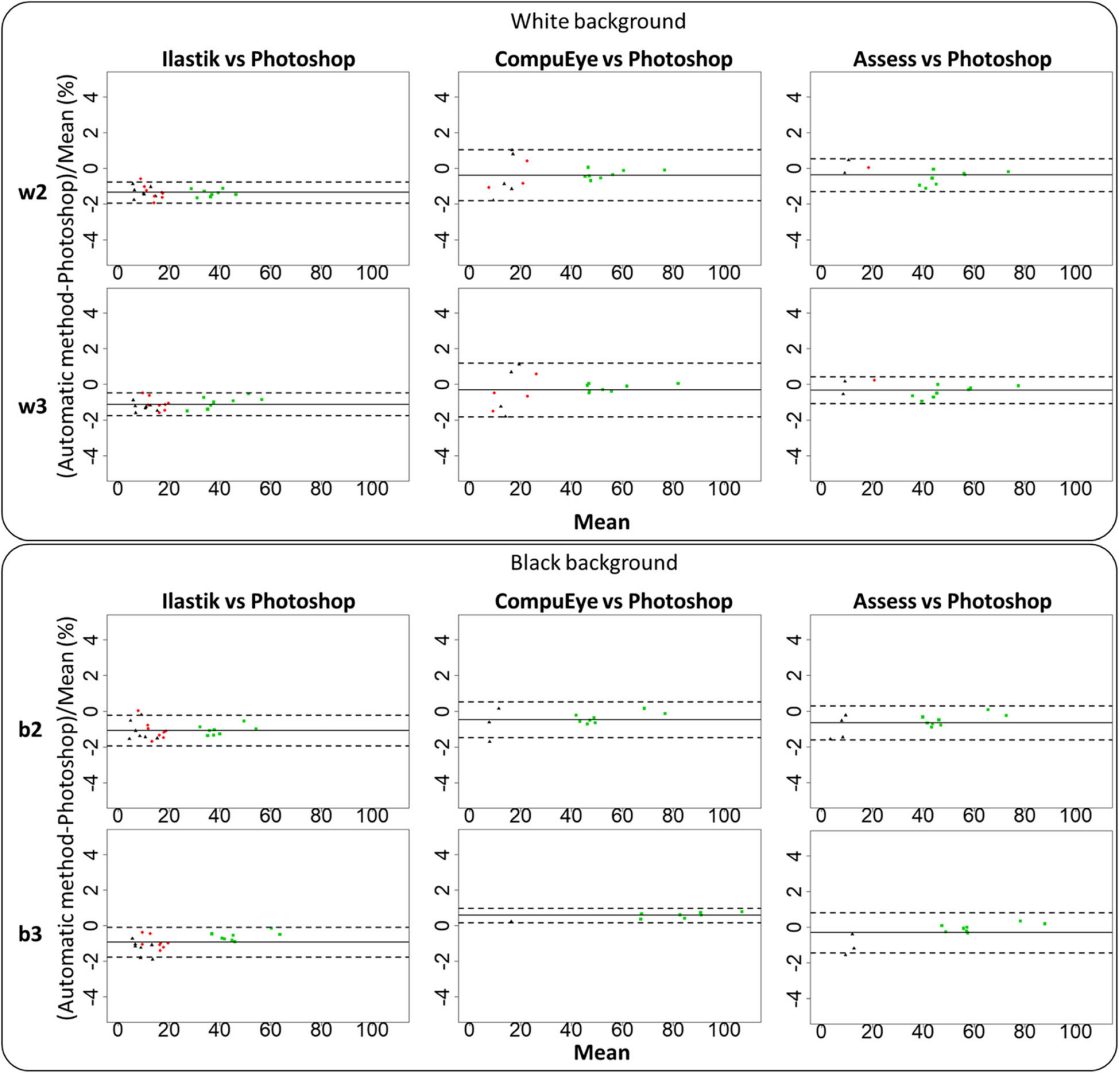
Bland-Altman plots of estimated damage area (automated method vs Photoshop). The central solid line indicates the mean difference, the two outer dashed lines indicate ±1.96 standard deviations. The observations that correspond to each *A. thaliana* genotype are shown in different colors and shapes. Black triangles indicate Bla-2, red dots indicate Col-0 and green squares indicate Kon. Data were obtained from *A. thaliana* Col-0, Bla-2 and Kon genotypes, infested with 20 *T. urticae* adults for 4 days; *n* = 8. Lighting conditions (brightness, contrast for each case): w2 and b2 = Automatic threshold (30,-20 White; 40,-10 Black); w3 and b3 values were selected for each background adding 10 values of brightness, maintaining contrast values

**Fig. 7:**
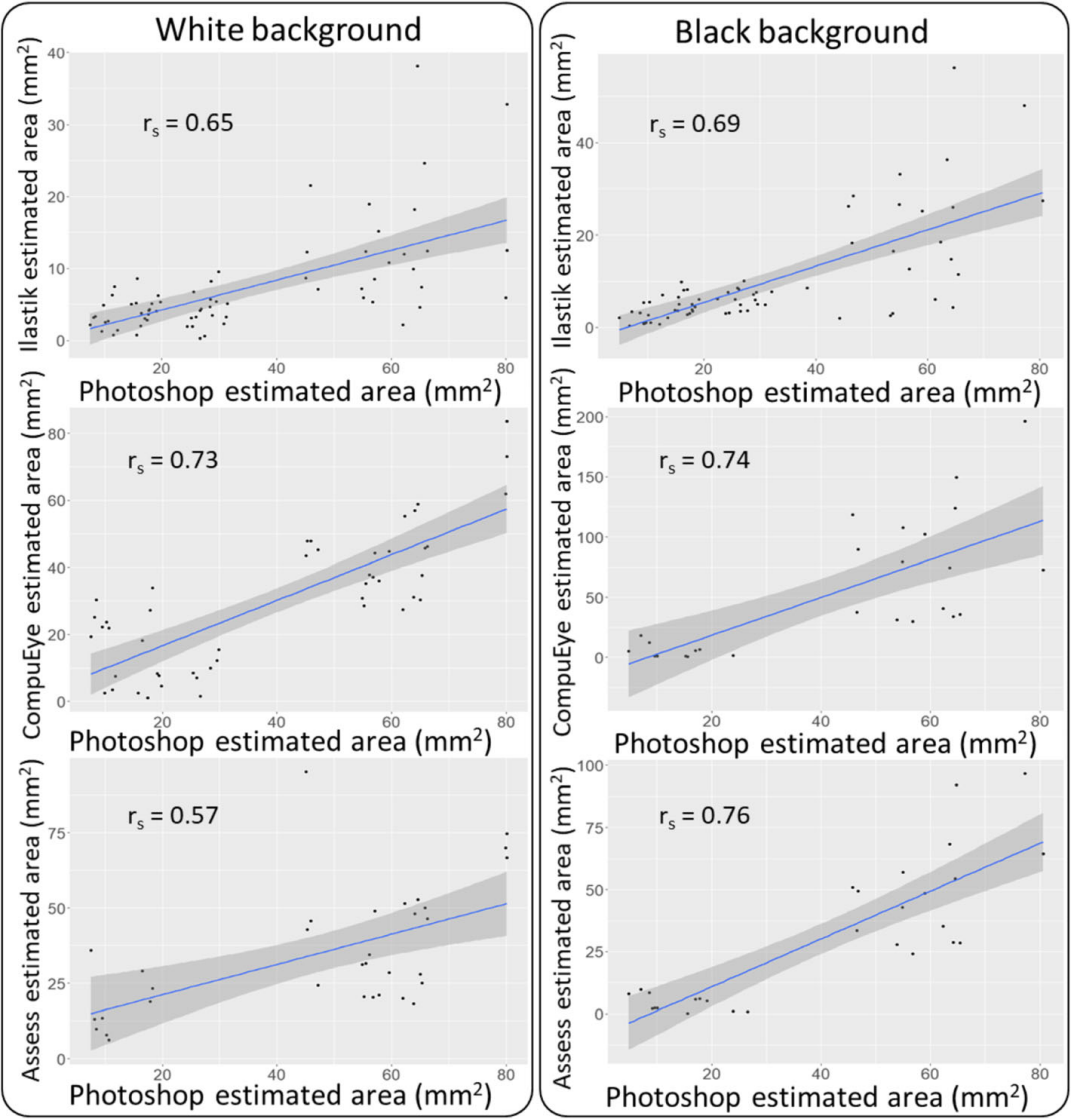
Spearman correlation coefficient (r_s_) describing the relationship between the damage estimated areas by Photoshop and by the automatic methods. The blue line represents the best fit and the grey areas show a confidence interval with 95% probability

**Fig. 8:**
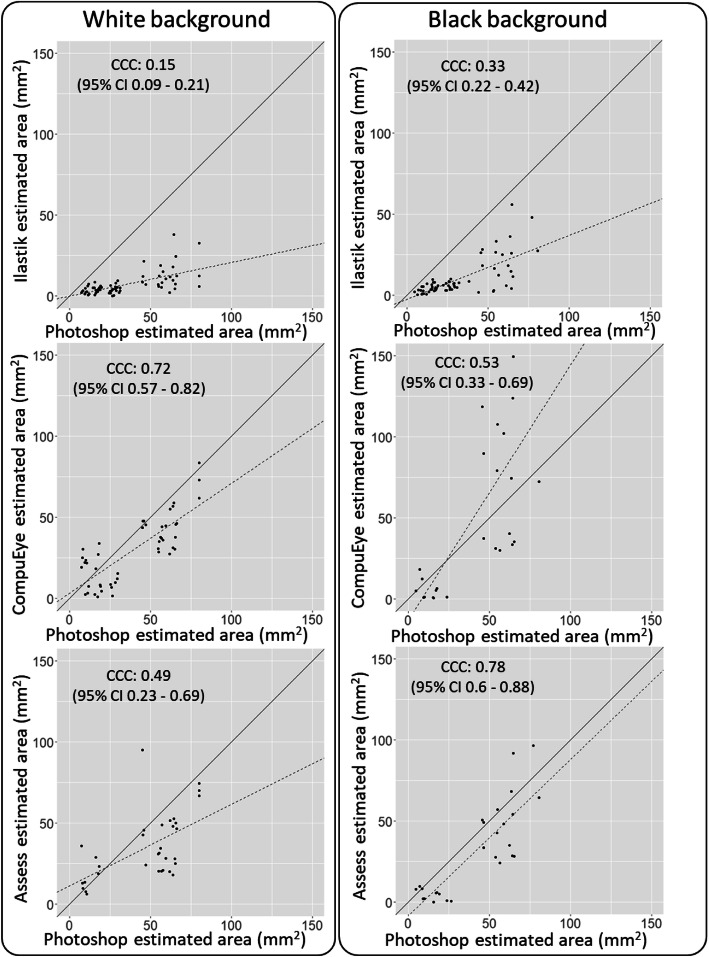
Lin’s concordance correlation analysis of *T. urticae* damage estimated manually (Photoshop) versus automatic methods. The solid line represents the line of concordance, indicating perfect agreement among methods. The dotted line indicates the line of the best fit to the values. The coefficient (CCC) is represented for each case and its respective 95% confidence interval (CI)

B&A plots were produced to study the agreement of each damage quantification method with the Photoshop standard procedure (Fig. 6). The differences were expressed as percentages of the values [(Method A – Method B)/mean)]. Due to the poor accuracy of the results on the conditions w1 and b1, the plots were only produced for the conditions w2, w3, b2 and b3. More replicates were analysed in the Ilastik-Fiji/manual annotation plots because less samples were lost during control correction. All the methods were biased to identify less damage compared to the standard, with the exception of CompuEye under the condition b3 on a black background (Fig. 6). The aforementioned exception was also the only case in which CompuEye presented narrower limits of agreement compared to the rest of the methods. The agreement limits for Ilastik were generally the narrowest (Fig. 6), followed by those from Assess, which indicated more consistency for these two estimation procedures. However, Ilastik bias to identify less damaged areas was larger compared to the other two methods. On average, Ilastik detected up to 1.36% (28 mm^2^) less damaged areas than Photoshop, while Assess detected up to 0.37% (17 mm^2^) less area and CompuEye overestimation reached 0.58% (53 mm^2^) (Fig. 6). Agreements between the automatic and the standard methods were also analysed by calculating Lin’s concordance correlation coefficient (CCC) and Spearman correlation coefficient. While the correlation coefficient identifies relationships among methods, Lin’s CCC is also able to detect constant bias and penalise accordingly. Regarding the Spearman coefficient, the correlation was significant (*p* < 0.001) for all the methods analysed. The correlation with the reference method was higher when the images were taken on black background for all the automatic methods (Fig. 7). When analyses were performed using Lin’s CCC, no perfect relationship was detected between the automatic methods and the manual standard approach. Except for CompuEye on the black background (Fig. 8), the rest of the methods displayed a tendency to underestimate damage compared to the standard. The highest CCC occurred for Assess on a black background (Fig. 8), indicating a better agreement, although most of its values were below the line of concordance. As expected from the previous B&A plot results, Ilastik had the lowest CCC values for both backgrounds, although were higher in the black background. This method was highlighted as the one with the largest bias in the B&A plots (Fig. 6), and since the CCC penalises this tendency, its values were the lowest.

### Comparative analysis of damage detection on control rosettes

**Fig. 9:**
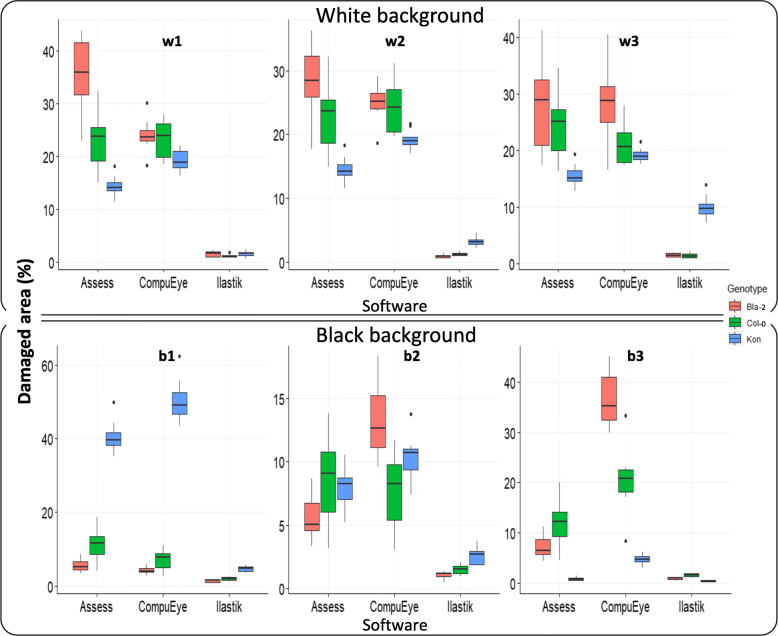
Box-and-whiskers plots of the damaged area percentage identified on control rosettes. Each automatic method is assessed under different lighting conditions and on white (top row) and black (lower row) backgrounds. Data are obtained from *A. thaliana* Col-0, Bla-2 and Kon genotypes, n = 8. Lighting conditions (brightness and contrast for each case): **w2** and **b2** (central column) = Automatic threshold (30,-20 White; 40,-10 Black); **w1**, **w3**, **b1** and **b3** values are selected for each background subtracting and adding 10 values of brightness, respectively, maintaining contrast values. Black dots indicate outlier values

Due to the larger size of the control rosettes compared to the infested ones (t = 25.291, df = 1054.2, *p*-value < 0.001), confounding areas were more abundant in these rosettes. To control this effect, damaged areas in the control rosettes were expressed as a percentage of the total rosette area. Differences were identified when the percentages of damaged areas in the control rosettes were analysed using a GLM (Additional file 3: Table S4). The highest interactions indicated statistical differences for the percentages when genotypes were analysed under different lighting conditions and backgrounds (χ^2^ = 120.6, *p* < 0.001) (Additional file 3: Table S4). In general, damaged area values increased for the control rosettes when they were scanned on a white background (Fig. 9).

**Fig. 10:**
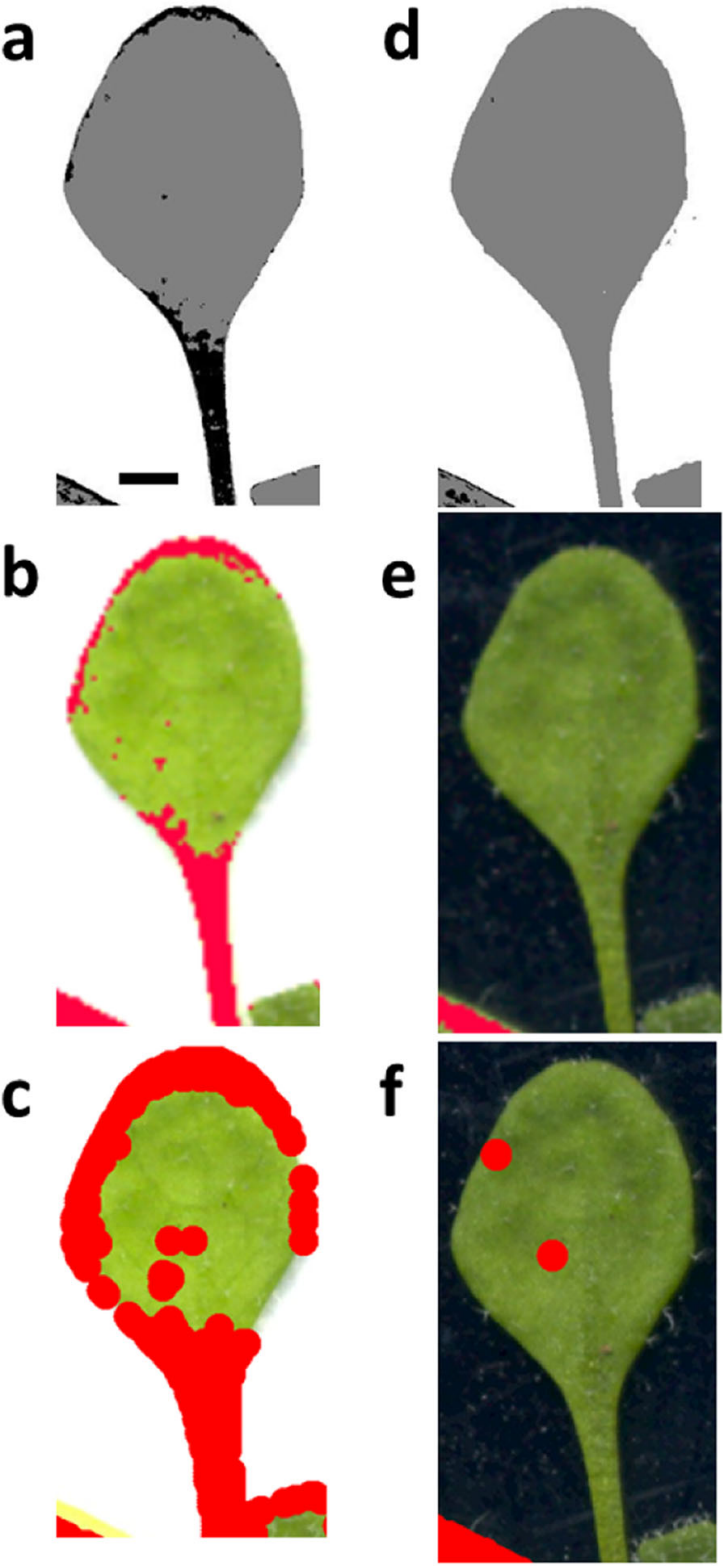
“Damaged areas” identified by the automatic methods on control rosettes. Identified areas are compared on a leaf among the three automatic methods as an example. A white background (**a**-**c**) and a black background (**d**-**f**) are used for comparison. Damaged areas are identified by Ilastik in black (**a**, **d**). Red colour identifies damaged tissue by Assess (**b**, **e**) and by CompuEye (**c**, **f**). Black scale bar indicates 1 mm

The lighting variations on each of the backgrounds did not cause statistically significant differences on the damaged areas (Additional file 3: Table S5). Assess and CompuEye had a tendency to detect more basal damage for Bla-2 and Col-0 genotypes on the white background, compared to Kon. This behaviour on genotypes that normally presented low damage area values for the infested plants, led to the loss of information when the control correction was applied. CompuEye was the software that identified more damage on control rosettes (up to 50%), followed by Assess (up to 40%). The post-hoc analysis highlighted Ilastik as the method that identified less damaged areas on the control rosettes (up to 10%, Additional file 3: Table S5). Errors, such as the identification by CompuEye and Assess of the petioles as damage (Fig. 10), were controlled under the black background.

## Discussion

In the last years, great efforts have been done to improve the understanding of the molecular bases of Arabidopsis resistance to spider mites [10–16]. Since the screenings to evaluate plant resistance require an appropriate method for plant damage quantification, most researchers of the spider mite community have used a protocol to quantify leaf damage based on manual annotation [7]. This procedure has allowed the identification of genes involved in Arabidopsis defence against spider mite, their role in defence against spider mites in crops, and the existence of mite adaptations to different plant hosts [26–29]. However, this method has some weak important points. It is subjective, dependent on the human eye, time consuming, and presents high inter and intra-rater variability. In this context, to establish an alternative procedure for an efficient and automatic quantification of spider mite damage in Arabidopsis using whole rosettes was required. Among the different automatic programs already described to quantify plant damage, Assess, CompuEye and Ilastik methods were selected because they had been previously used for the quantification of similar plant subtle symptoms [21–25]. Different conditions and parameters were tested to fix the optimal settings for the establishment of a reliable automatic method with high accuracy, precision and concordance to be routinely used in the *A. thaliana-T. urticae* interaction studies.

### Background and brightness

It is well known that the scanning conditions and the image quality affect the quantification of the damage calculated by digital methods [18, 23, 30]. These analyses require specific thresholds, training and sensitivities for a correct identification and quantification of the damage [31, 32]. In Arabidopsis, the leaf symptoms due to the spider mite feeding are identified as chlorotic spots or small regions in pale yellow or white colour [6], which are highlighted on a white background. Indeed, the manual method used as reference generally detected more damage when the measurements were done on a white background. This feature suggests that the bright surrounding the plant tissues alters the detection of damage achieved by the human eye. Likewise, our findings supported that the Assess and CompuEye programs also recognised greater damage in the control infested rosettes when the images were taken on a white background. The brightest conditions produced the highest capacity to detect damaged tissues but, at the same time, the highest error rates due to colour similarities and reflection [18, 32]. Consequently, the overestimation of the spider mite damage in the control rosettes caused the loss of data when the correction was applied, and rendered whole genotypes without any information after infestation. To elude an excess of errors and to avoid the misinterpretation between mite damage, trichome-rich regions and early senescent symptoms, the acquisition of images on a black background was also tested. Under these conditions, the estimation of the damaged area in the control rosettes by the Assess and CompuEye programs was significantly lower than the values determined on the white background, avoiding known errors such as the identification of petioles as damage [33, 34]. In addition, the importance of lighting conditions on image analysis pushed us to assess the combination of contrast and brightness in the quantification of the plant damage. As expected, extreme conditions of brightness were hurdles for the automatic methods [30]. In accordance to Kirk et al. [35], a subset of moderate settings rendered the most reliable and robust results. These settings were the automatic condition proposed by the scanner and 10 points of brightness variations above and below that value. The analysis of these three lighting conditions provided a mix of results for each background and the Ilastik program was the one that generated less variable results with a higher reproducibility for most of the conditions tested.

### Accuracy, precision and concordance

Accuracy refers to closeness of a measurement to a specific value while precision denotes the closeness of the measurements to each other. Both concepts are mandatory in the evaluation of any automated method based on the application of computer image processing to quantify plant damage. The closeness to the standard was greatly affected by the dimmer conditions independently of the background. Therefore, a brightness reduction under the automatic detection was not advisable, as previously reported [36]. In our study, the CompuEye method performed worse under conditions where the other programs had positive results. It failed to reproduce resistance ranges among the Bla-2, Col-0 and Kon accessions on the white background and lost data from some genotypes on the black background due to excessive damage estimation on the control rosettes. Besides, it had wider limits of agreement on the B&A plots, although its bias were the lowest. These results agreed with the high variability of its estimations. It was also the only method that incurred in overestimation, and the most affected by changes on lighting and background conditions. The main hurdle in the Assess method was the estimation of damage for the control Col-0 rosettes. This method consistently identified more damage in several Col-0 control plants than in infested plants, leading to loss of data. Furthermore, the values of damage obtained by this method on a white background for the Kon accession were the most variable. These results agreed with the fact that the RGB model in which they rely was susceptible to light variations [33]. On the contrary, the variability found by the Ilastik method was generally similar to the standard values except for the Kon genotype, whose highly variable data were detected independently on the condition and the software used. Ilastik results also consistently reproduced the Kon > Col-0 *~* Bla-2 relationship regardless of the condition. The proximity of the aforementioned values to the reference ones, the small standard deviation, and the uniformity of the relationship among genotypes identified the brightest condition tested on the black background (condition b3) as the best condition for the Ilastik program. The quotient of mean damage Kon/Col-0 by the Ilastik method was 4.5 for the condition b3, similar to the results reported by Santamaria et al. [13, 14]. The reproducibility of the relation in the damage found in Kon/Col-0 was a good indicator of the Ilastik reliability.

In terms of concordance, which defines the similarity, harmony or consistence between two different measurements, the Assess and CompuEye methods agreed more to the reference method. However, the wider agreement limits for the Assess and CompuEye procedures suggested a higher variation between their estimated areas compared to Ilastik, the latter having noticeably a higher underestimation bias. Likewise, although the correlation coefficients were significant for all the methods, Lin’s CCC values were superior for the Assess and CompuEye methods. As CCC evaluated agreement between the two methods by analysing the deviation of their relationship from a line that goes 45° through the origin [37], CCC values for the Ilastik method were heavily penalised due to its bigger bias. The close relationship detected by the correlation analysis and CCC between Assess, CompuEye and the standard method could be due to their similar strategy to detect damaged areas.

**Fig. 11:**
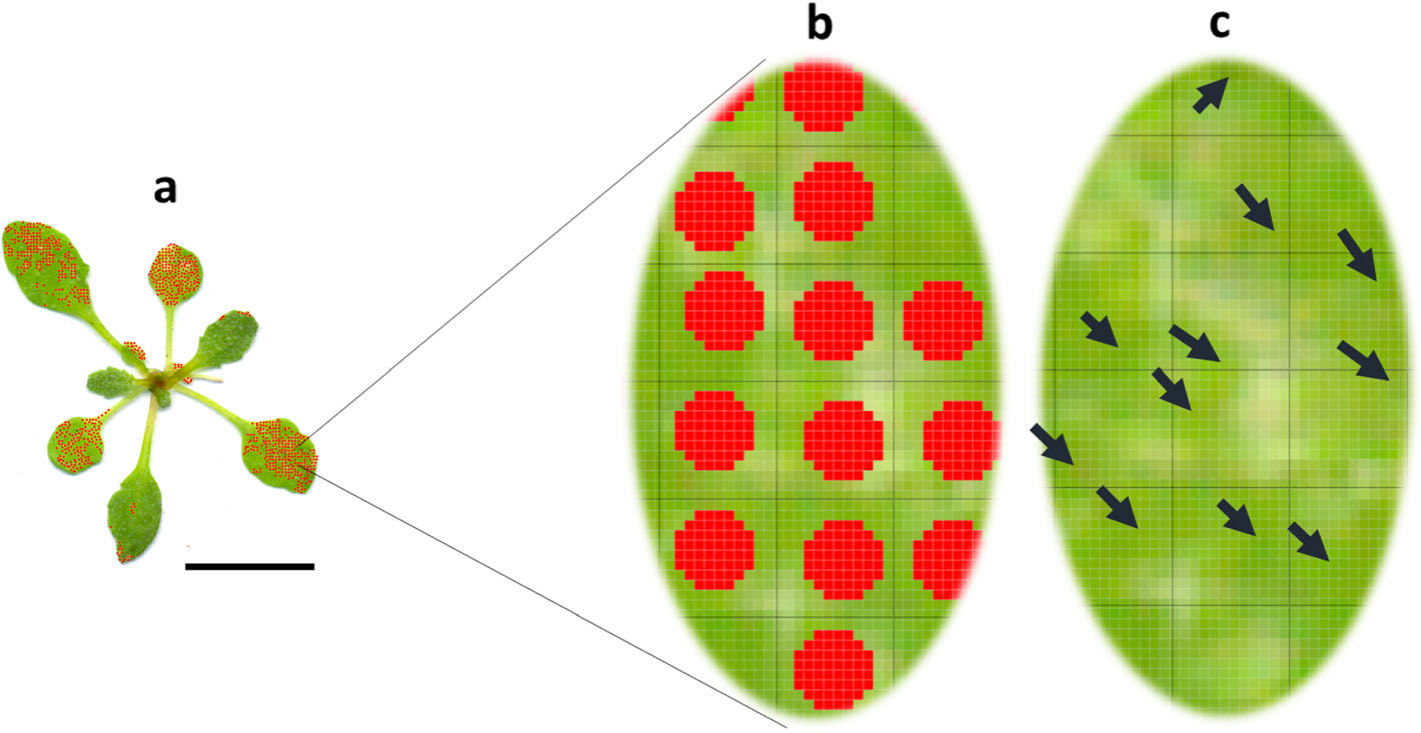
Analysis of the overestimation phenomenon that occurs in the Photoshop method. A random region of a rosette processed by the standard method (**a**) is zoomed (**b**). Healthy tissue can be seen, as indicated by black arrows (**c**), inside the squares assumed by the method to be filled by chlorotic tissue. Black scale bar indicates 1 cm. The sides of the squares on **b** and **c** are 0.25 mm

The standard method involved the manual segmentation, by means of red dots, of the damaged regions that were going to be later extrapolated and summed as squared areas of 0.25*0.25 mm [7]. Apart from the variations on the measurements produced by the subjectivity or rater experience [38, 39], this method also incurred in an intrinsic overestimation related to the selection procedure. When a region was selected as damaged, a mixture of healthy and chlorotic tissue was often included in the selection (Fig. 11). This phenomenon occurred inside of the damaged areas but was more frequent in the boundaries of the damaged regions (Fig. 11c). As a consequence, healthy tissue was counted as damaged. The miscounting increases every time that a new selected square had these characteristics, which happened with moderate frequency. The rate of miscounting also proliferated as the total damage increased, especially if the areas were spread. On susceptible genotypes such as Kon, where the damaged areas were extensive and dispersed, the incidence was higher.

The Assess and CompuEye methods behaved similar to the manual method performed on Photoshop, tending also to overestimate the damaged areas and because of that, their concordance was higher. The segmentation procedure on the Classic Threshold Panel from the Assess software makes use of a simple thresholding procedure by choosing a range of pixels inside a selected colour space [40, 41]. The selection has two fixed boundaries that encompasses the pixels regarded as of interest. However, due to the complexity of a rosette, the damaged areas included separated ranges of pixels and not necessarily a single range. When a single broad range was selected, healthy tissue was also included in the selection such as young leaves, trichome-rich regions and leaf borders. The extent of misidentification depended on the presence of confounding areas, which vary among rosettes. On the other hand, the CompuEye segmentation procedure also relies on a simple thresholding procedure. The available systems are pre-programmed to identify lack of green colour and to increase their scope by regulating their sensitivity [23]. As in the manual method, the image is divided in square units of a selected size and the identification procedure analyses each square at a time, calculating an average colour [42]. The strategy of choosing a fixed colour/pixel-value as a threshold below which damage selection is done, works similar to the Assess procedure and carries the same drawbacks.

Different from the two previous automatic techniques, the Ilastik method uses a more complex approach to identify damage, which is more than likely the reason of its low concordance [43]. It allows the identification of pixels as damaged areas in complex texture images by combining active learning and machine learning techniques [44]. The identified pixel information provided by the user is initially bootstrapped. Subsequently, the input is used for the construction of individual decision trees, and the pixel by pixel classification of the whole image occurs by means of a random forest classifier [44, 45]. Classification by means of random forest is deemed as one of the most efficient learning machine techniques and has been reported as robust when trained using small sample sizes [46, 47]. Moreover, when used for the identification of biological stresses on plants, it has been identified as superior compared to other supervised machine learning techniques [48]. The software also guides users to ambiguous regions to fine-tune the classification by adding extra information. Instead of a fixed area or array of pixels, as the previous strategies used by Assess and CompuEye, the Ilastik procedure allows to selectively pick those pixels that better represent what the user identifies as damaged tissue. This sort of selection allows a finer estimation of damage on a complex visual environment such as a rosette image. Also, all the previous procedures occur in a user-friendly interface that does not require any programming knowledge from the user.

Some of the main limitations of the automatic procedures found during the development of the present study were related to their sensitivity to light and background conditions. Therefore, the present study identified those lighting and background conditions where the programs tended to have their best outcome. The high sensitivity of the Ilastik method highlighted as the best choice. However, it prevented the use of the trained model on certain Arabidopsis ecotypes to identify damage on another one, which led to the training of individual models for each ecotype tested. As a machine learning based software, Ilastik requires training data to be able to effectively identify damage from healthy tissue. This is a challenge for the present experimental design due to its replication limitation. However, in the array of available artificial intelligence technologies, the strategy used by Ilastik optimises the usage of available training data, reducing the total amount required, as discussed below.

### Ilastik as a machine learning approach

As stated before, the Ilastik background algorithm that identifies the damaged areas in the rosettes is deemed as supervised machine learning. The capacity of this technology to identify patterns makes it useful in scientific tasks, such as the detection and measurement of disease symptoms by means of specific algorithms [49]. Software running the afore mentioned technology also improve when given new information in an automated fashion, regardless of the knowledge of the underlying model for the raw data [50]. Among machine learning techniques applied to image analysis, deep learning methods are alternatives to the supervised machine learning employed by Ilastik. They use an important number of images provided by the user to identify pixels. Therefore, this alternative explores high amounts of raw images and provide labels and associate image regions with them without human intervention [51]. Deep learning approaches for image analysis, such as convolutional neural networks, are very powerful. However, they require large amounts of training data, being precisely the decrease of the training data size one of the main areas of research for this methodology [52].

On the other hand, supervised machine learning involves algorithm parametrization by means of human guidance, allowing the extraction of meaningful patterns from the samples provided [53]. Unlike deep learning, the machine learning methodology used by Ilastik provides classification conclusions based on significantly less image requirements. Image replication depends on the number of rosettes cultured into growth chambers, whose capacity is generally limited. Additionally, Arabidopsis rosettes must be infested manually one by one using tens of minuscule mites, which have to be removed also by hand prior to the scanning procedure. As a consequence, phenotyping experiments are generally in the range of 6 replicates [13, 14]. This is a strong challenge to the use of deep learning techniques and renders their use unpractical, since the training phase requires hundreds to thousands of images [54]. Dissimilar to deep learning techniques, the strategy employed by Ilastik reduces the feature space to the user-selected pixels, using the training data only to identify the decision surface [52]. As a consequence, this method allows a balance between accuracy, simplicity and speed, and significantly reduces the amount of data required for the training and analysis processes [52]. Moreover, deep learning requires a heavy processing power and a large amount of time for the training phase, and usually involves programming abilities [54, 55]. Since one of the main goals of the present work is to provide an accessible and reproducible method, these requirements would also act as a restrain.

Another advantage of the machine learning technique used by Ilastik is that of improving its performance when more information is given. In that regard, future tests will be done to validate and enhance this methodology. Issues that should be checked are the results obtained upon enrichment of the training samples, the error reduction by means of the control images and the expert-segmented images, and the maintenance of the relationship among the damaged areas in different Arabidopsis ecotypes. Moreover, due to the plasticity of the supervised machine learning methodology, the current protocol can also be applied to the identification of damage from different stresses such as fungal, viral or bacterial infections. Likewise, the present protocol could also be transferred to other plant species, expanding further its applicability spectrum. As future approaches that could be applied to the present damage identification requirements are emerging deep learning strategies such as data augmentation, transfer learning, or domain adaptation. The aforementioned machine learning approaches make use of diverse strategies in order to ameliorate training data requirements while maintain efficacy and specificity. This is a feature that render their use appealing due to the image availability restriction of the present studies.

## Conclusions

In this work, a new and automatic method of damage quantification produced by spider mite feeding in Arabidopsis whole rosettes is proposed. Our findings are a clear advance on the available manual method currently accepted to quantify damage in infested plants. Among the methods tested, the combination of Ilastik and Fiji used in tandem is the most accurate, precise and reproducible procedure to quantify spider mite damage in Arabidopsis rosettes. The concordance analysis shows a bias for the results obtained by the Ilastik procedure that reveals the overestimation of damage produced with the rest of approaches, including the reference one. Besides, the study of the influence of the image scanning conditions in the damage quantification enabled to select the proper lighting and background conditions to evaluate plant damage under the identified system. Thus, this automated method based on machine learning, non-subjective, not time consuming and with no inter and intra-rater variability can be considered as an enhanced and appropriate method to study different aspects of the plant-spider mite physiological interaction.

## Methods

### Plant material and growth conditions

Seeds for all the *A. thaliana* accessions were kindly provided by Dr. Vojislava Grbic (University of Western Ontario, Canada) and originally acquired from the ABRC (Arabidopsis Biological Resource Center). *A. thaliana* plants from the accessions Columbia (Col-0), Kondara (Kon) and Blanes (Bla-2) with different susceptibility to *T. urticae* [11] were used.

Seeds were planted and incubated for 5 days at 4 **°**C and plants were then grown in growth chambers (Sanyo MLR-350-H) under control conditions (23 **°**C ± 1 **°**C, > 70% relative humidity and a 16 h/8 h day/night photoperiod).

### Spider mite maintenance

A colony of *T. urticae* London strain (Acari: Tetranychidae), kindly provided by Dr. Miodrag Grbic (University of Western Ontario, Canada), was reared on beans (*Phaseolus vulgaris*) and maintained on growth chambers (Sanyo MLR-350-H, Sanyo, Japan) at 25 °C ± 1 °C, > 70% relative humidity and a 16 h/8 h day/night photoperiod.

### Infestation protocol

*A. thaliana* three-week-old plants were carefully infested with 50 or 20 *T. urticae* female adults per plant using a fine brush for the optimization of the conditions or for method comparisons, respectively. Three to eight replicates were used.

### Image acquisition and processing

After 4 days of infestation, mites were carefully removed from the rosettes and the entire rosettes were cut and scanned using HP Scanjet (HP Scanjet 5590 Digital Flatbed Scanner series). A millimetre paper was used as a size reference (Fig. 1a). To evaluate the effect of background and lighting variations in the damage estimation, images were taken on white and black backgrounds under six conditions: i) the lighting condition used by Cazaux et al. [7]; ii) the automatic brightness and contrast levels suggested by the scanning software; iii) two conditions that consisted on adding and subtracting 10 values of brightness to the automatic threshold, maintaining the contrast; and finally, iv) two extreme lighting conditions. All tested conditions are summarized in Table 4. Three biological replicates coming from three independent rosettes scanned at the same time were used as individual replicates.

**Table 4:** Scanning conditions employed to determine damage estimation stability using Col-0 genotype

Condition	Contrast	Brightness	Background
A1	−100	90	White / Black
A2	−50	25	
A3	−69	40	
A4	−69	30	
A5	−69	20	
A6	−56	1	

After the identification of the conditions that produced the lowest variability and the closest results to the standard, the accuracy, precision, reproducibility and concordance of the methods were studied, including the Bla-2 and Kon Arabidopsis genotypes in the analysis. These two aforementioned accessions are located at the opposite ends of the Arabidopsis susceptibility spectrum. Kon is the most susceptible and, consequently, the most damaged by spider mites, and Bla-2 the most resistant or less damaged by this phytophagous acari [11]. The previous information was considered important to evaluate the reliability of the methods tested. Images of the three Arabidopsis, Col-0, Kon and Bla-2 genotypes were taken under the lighting optimal conditions in black and white backgrounds. Tested conditions are summarized in Table 5. Eight biological replicates coming from eight independent rosettes scanned at the same time were used as individual replicates.

**Table 5:** Scanning conditions employed to test the performance of each method using Bla-2, Col-0 and Kon

Condition	Contrast	Brightness	Background
W1	−20	20	White
W2	−20	30	
W3	−20	40	
B1	−10	30	Black
B2	−10	40	
B3	−10	50	

All the images were taken in Adobe RGB colour mode, with 1200 dpi of resolution and saved as tiff files. Individual rosettes were separated from the original images (duplicated/renamed) using Adobe Photoshop program, retaining the size and resolution, and were saved as tiff files (Fig. 1b).

### Damage quantification

Plant damage was identified as the total area of chlorotic spots detected after spider mite feeding. Image processing and quantification of the feeding damage was performed with Adobe Photoshop CC 2018 v20.0 [56], APS Assess v2.0 [40], Compu Eye (http://www.ehabsoft.com/CompuEye/LeafSArea/) [23] or Ilastik 1.1.3 (https://www.ilastik.org/) [44] followed by the Fiji software (https://fiji.sc) [57]. The manual identification of injured areas was done by the Photoshop program according to Cazaux et al. [7], and used as the standard method to compare the results with the automatic procedures. Briefly, a layer was created over the original rosette image and a grid of equal square sizes was applied as a guide. Damaged areas were manually identified by covering them using dots. Each dot corresponded to a small square formed by the grid. The final number of dots was calculated and multiplied by the area of the squares to obtain the total damaged area.

For the three automatic methods, a summary of the main followed steps is depicted in the Additional File 4. CompuEye software was used according to Bakr [23]. In short, the first step was to calibrate the system using the millimetre paper guide scanned with the rosettes. Each of the four available systems and sensitivities of the software were tested for every genotype, at different light conditions and backgrounds. The custom detection system was also tried. The system/sensitivity combination that better matched the highlighted areas in Photoshop was recorded and used to batch process the images. In the case of the APS Assess software, previous protocols [58–60] were optimized to specifically detect spider mite plant damage. All the analyses were performed using the Classic Panel, and the combination of colour plane and pixel range that reproduced better the standard areas was selected. Macros were programmed for batch processing the images of each genotype under different lighting conditions and backgrounds.

A modification of the method used by Visschers et al. [24] was also tested. The aforementioned method uses Ilastik as an interactive machine learning tool to segment images [52]. This software was trained using images composed of control and treated rosettes to identify damaged tissue. Training was done for each genotype scanned on each specific lighting condition and background, from which six to seven images of damaged and control rosettes were randomly selected. During the training phase, the pixels corresponding to background, healthy tissue and damaged areas were labelled. The characteristic of the software to instantly model the unlabelled regions of the image, allowed the evaluation of the classification performance by using the unlabelled pixels as testing datasets. Incorrectly identified areas by the program were amended, which allowed a re-training procedure based on the increased amount of training labels. Uncertain areas indicated by the software were annotated to help the robustness of the results. The previous steps were repeated and information was given to the software during the training procedure until no significant improvement was detected in performance. Further evaluation occurred by comparing the results to the manual segmentation done by a specialist on the same images. Using the information from the training procedure, the images were opened in Ilastik (Fig. 1c), batch segmented (Fig. 1d), and exported to Fiji as 8-bit images (Fig. 1e). Then, images were reopened in Fiji and converted to black and white (Fig. 1f). To discriminate damage from noise, the damaged area was corrected with a cluster size threshold selected from control rosette cluster frequency graph (Fig. 1g). Macros were designed and the images batch processed. The protocol of spider mite damage quantification in Arabidopsis rosettes using Ilastik-Fiji tandem is detailed in Additional file 5. The training files are available in the website of our research centre (http://www.cbgp.upm.es/files/Ilastik_paper.php).

### Data correction

After the damage quantification by the automatic methods, values were corrected using the damage identified in the controls without mites. Non-infested plants were used to assess the misidentification of damaged tissue that is normally expected due to the visual complexity of a rosette. To do so, the control rosettes of each genotype were analysed under the same thresholds and conditions that were used on their treated counterparts. The percentage of damaged tissue on each control plant was calculated based on the damaged area and the total rosette area. A mean percentage was calculated from the previous values that was multiplied by the total area of each infested rosette. Uncorrected values were then modified by subtracting the multiplication of the mean percentage by the value of each total rosette area. The mean of the damaged areas detected in the control rosettes was calculated by using the following formula:

$$ {\overline{x}}_c=\frac{\sum \left({DA_c}_i/{TA_c}_i\right)}{n} $$

${\overline{x}}_c$ accounts for the mean of the percentages of damaged area in the control rosettes; *DA*_*ci*_ indicates the damaged area in each independent rosette (*i*th); *TA*_*ci*_ represents the total area of the *i*th rosette; and *n* accounts for the number of replicates. The corrected data were calculated by:

$$ {A}_i=D{A_t}_i-{\overline{x}}_c\ast {TA_t}_i $$

*A*_*i*_ accounts for the corrected damaged area of the *i*th rosette; *DA*_*ti*_ represents the uncorrected damaged area of the *i*th rosette; and *TA*_*ti*_ the total area of the treated *i*th rosette.

The previous correction procedure was performed independently for each genotype under each lighting/background condition tested.

### Statistical analyses

To evaluate the performance of the automatic methods and to compare them with the standard manual method, the concepts of accuracy, precision, reproducibility, concordance and bias were evaluated [18, 61]. Data from the optimization of lighting conditions were analysed by a Generalized Linear Model (GLM). The aforementioned test was performed with a gamma distribution and an inverse link function to analyse lighting and background effects using the model Damage~Program*Background*ConditionID. Reproducibility was tested by changing the lighting conditions and background and the data assessed by a GLM with exponential distribution and an inverse link function, applying the best fit model Damage~Program*ConditionID*Genotype. Bland-Altmann (B&A) plots were also used to visually identify variability along with Coefficients of Repeatability (CR). Concordance was compared by using B&A plots expressing the differences as percentages of the values. Agreement was also showed by calculating Lin’s Concordance Correlation Coefficient (CCC) and Spearman Correlation Coefficient (SCC). Damage identified by the automatic methods in the control rosettes was expressed as percent of the total area and compared using a GLM analysis with a gamma distribution and inverse link function in the best fit model DamagePercent~Program*ConditionID*Genotype. A Chi-Square distribution test was used to analyse all the GLM models. Differences were identified by the application of a pair-wise comparison analysis with Bonferroni correction. Rosette area for control and infested plants were compared by t-test. For all tests, results having *p* values ≤0.05 were considered as significant. All statistical and data analysis was performed using the R software version 3.5.3 [62].

## Supplementary information

**Additional file 1: Table S1.** Pixel threshold ranges used to identify damage by Assess 2.0. **Table S2.** Combination of detection system and sensitivity used to identify damage by CompuEye. **Table S3.** Descriptive statistics for the estimated damaged areas identified on different backgrounds and lighting conditions. **Table S4.** Descriptive statistics for the estimated damaged areas identified on different backgrounds and lighting conditions using Bla-2, Col-0 and Kon.

**Additional file 2: Macro S1.** Assess macro used to select area of interest and identify damaged areas. **Macro S2.** Fiji macro used to transform grey scale images to black/white. **Macro S3.** Fiji macro used to identify the pixel cluster size. **Macro S4.** Fiji macro used to calculate damaged areas on white/black images. **Fig. S1.** Frequency of appearance of damage clusters in Bla-2, Col-0 and Kon control rosettes.

**Additional file 3: Table S1.** Statistical R output for GLM analysis. The test compares the damaged areas identified by the automatic methods under six lighting conditions and white/black backgrounds for the Col-0 genotype. **Table S2.** Statistical R output for GLM analysis. The test compares the damaged areas identified by the automatic methods under three lighting conditions and white/black backgrounds for the genotypes: Bla-2, Col-0 and Kon. **Table S3.** Pair-wise comparison with Bonferroni correction comparing method results under different lighting conditions. The test compares the damaged areas identified by the automatic methods under three lighting conditions on white/black backgrounds for the genotypes: Bla-2, Col-0 and Kon. **Table S4.** Statistical R output for GLM analysis. The test compares the damaged areas identified by the automatic methods under three lighting conditions and white/black backgrounds for the controls of the genotypes: Bla-2, Col-0 and Kon. **Table S5.** Pair-wise comparison with Bonferroni correction comparing control rosette results under different lighting conditions. The test compares the damaged areas identified by the automatic methods under three lighting conditions on white/black backgrounds for the control of the genotypes, Bla-2, Col-0 and Kon.

**Additional file 4.** Flow chart depicting the general steps for the three automatic analysis tested.

**Additional file 5.** Detailed protocol for the spider mite damage quantification in Arabidopsis whole rosettes.

## Data Availability

All relevant supporting data sets are included in the article, its supplemental files and http://www.cbgp.upm.es/files/Ilastik_paper.php. The datasets used and/or analysed during the current study are available from the corresponding author on reasonable request.

## Abbreviations

APS: American Phytopathological Society
Macros: Macroinstructions
GLM: Generalized linear model
SD: Standard deviation
CV: Coefficient of variation
Kon: Kondara
Bla-2: Blaines-2
Kon: Kondara
Col-0: Columbia-0
B&A: Bland–Altman
CR: Coefficient of repeatability
CI: Confidence intervals
CCC: Concordance correlation coefficient
RGB: Red Green Blue
SCC: Spearman correlation coefficient
