# Supplementary material for: Saving time maintaining reliability: a new method for quantification of Tetranychus urticae damage in Arabidopsis whole rosettes
Source: BMC Plant Biol. 2020 Aug 27;20:397. doi: 10.1186/s12870-020-02584-0 (PMC7450957; doi:10.1186/s12870-020-02584-0)
Supplement: Supplementary file 1 — Additional file 1: Table S1. Pixel threshold ranges used to identify damage by Assess 2.0. Table S2. Combination of detection system and sensitivity used to identify damage by CompuEye. Table S3. Descriptive statistics for the estimated damaged areas identified on different backgrounds and lighting conditions. Table S4. Descriptive statistics for the estimated damaged areas identified on different backgrounds and lighting conditions using Bla-2, Col-0 and Kon. [file 12870_2020_2584_MOESM1_ESM.pdf]

Table S1. Pixel threshold ranges used to identify damage on Assess 2.0. Ranges were identified by analysing the genotypes: Bla-2, Col-0 and Kon under different lighting and background conditions.

| Genotype | Background | Lighting condition | Rosette | | Damaged area | |
|---|---|---|---|---|---|---|
| | | | Colour plane | Pixel range | Colour plane | Pixel range |
| Bla-2 | White | W1 | HSV/S | 80;255 | HSV/V | 190;255 |
| | | W2 | HSV/S | 80;255 | HSV/V | 205;255 |
| | | W3 | HSV/S | 80;255 | HSV/V | 218;255 |
| | Black | B1 | HSV/V | 80;255 | HSV/V | 165;255 |
| | | B2 | HSV/V | 80;255 | HSV/V | 175;255 |
| | | B3 | HSV/V | 80;255 | HSV/V | 185;255 |
| Col-0 | White | W1 | HSV/S | 80;255 | HSV/V | 180;255 |
| | | W2 | HSV/S | 80;255 | HSV/V | 190;255 |
| | | W3 | HSV/S | 80;255 | HSV/V | 200;255 |
| | Black | B1 | HSV/V | 80;255 | HSV/V | 155;255 |
| | | B2 | HSV/V | 80;255 | HSV/V | 167;255 |
| | | B3 | HSV/V | 100;255 | HSV/V | 175;255 |
| Kon | White | W1 | HSV/S | 80;255 | HSV/V | 190;255 |
| | | W2 | HSV/S | 80;255 | HSV/V | 200;255 |
| | | W3 | HSV/S | 80;255 | HSV/V | 210;255 |
| | Black | B1 | HSV/V | 80;255 | HSV/V | 148;255 |
| | | B2 | HSV/V | 80;255 | HSV/V | 158;255 |
| | | B3 | HSV/V | 80;255 | HSV/V | 168;255 |

Lighting conditions (Brightness, Contrast for each case): W2 and B2 = Automatic threshold (30,-20 White; 40,-10 Black); W1, W3, B1 and B3 values were selected for each background subtracting and adding 10 values of brightness, respectively, maintaining contrast values.

Table S2. Combination of detection system and sensitivity used to identify damage on CompuEye. Ranges were identified by analysing the genotypes: Bla-2, Col-0 and Kon under different lighting and background conditions under the automatic unit tuner value.

| Genotype | Background | Lighting condition | System | Sensitivity |
|---|---|---|---|---|
| Bla-2 | White | W1 | 3 | 13 |
| | | W2 | 3 | 12 |
| | | W3 | 3 | 11 |
| | Black | B1 | 2 | 0 |
| | | B2 | 2 | 0 |
| | | B3 | 2 | 0 |
| Col-0 | White | W1 | 3 | 16 |
| | | W2 | 3 | 15 |
| | | W3 | 3 | 13 |
| | Black | B1 | 2 | 2 |
| | | B2 | 2 | 0 |
| | | B3 | 2 | 0 |
| Kon | White | W1 | 3 | 15 |
| | | W2 | 3 | 14 |
| | | W3 | 3 | 13 |
| | Black | B1 | 2 | 6 |
| | | B2 | 2 | 3 |
| | | B3 | 2 | 3 |

Lighting conditions (Brightness, Contrast for each case): W2 and B2 = Automatic threshold (30,-20 White; 40,-10 Black); W1, W3, B1 and B3 values were selected for each background subtracting and adding 10 values of brightness, respectively, maintaining contrast values.

Table S3. Descriptive statistics for the estimated damaged areas identified on different backgrounds and lighting conditions.

| Condition \ Program | | Assess | | | CompuEye | | | Ilastik | | | Photoshop | | |
|---|---|---|---|---|---|---|---|---|---|---|---|---|---|
| | | Mean ± SD | Median | CV | Mean ± SD | Median | CV | Mean ± SD | Median | CV | Mean ± SD | Median | CV |
| White background | A1 | - | - | - | - | - | - | - | - | - | 47.30 ± 7.78 | 50.83 | 0.16 |
| | A2 | - | - | - | - | - | - | 59.92 ± 15.54 | 59.92 | 0.25 | 49.20 ± 8.56 | 51.29 | 0.17 |
| | A3 | - | - | - | - | - | - | 21.69 ± 14.06 | 18.29 | 0.64 | 48.56 ± 8.88 | 52.25 | 0.18 |
| | A4 | - | - | - | - | - | - | 33.19 ± 33.19 | 37.93 | 0.39 | 48.79 ± 7.65 | 50.6 | 0.15 |
| | A5 | - | - | - | - | - | - | 50.85 ± 8.94 | 55.2 | 0.17 | 49.38 ± 8.87 | 52.72 | 0.17 |
| | A6 | - | - | - | - | - | - | 540.71 ± 82.60 | 581.92 | 0.15 | 52.73 ± 8.22 | 56.01 | 0.15 |
| Black background | A1 | 43.34 ± 1.83 | 43.34 | 0.04 | 35.84 ± 26.83 | 47.27 | 0.74 | 5.43 ± 0.94 | 5.43 | 0.17 | 48.2 ± 6.75 | 48.87 | 0.14 |
| | A2 | 36.10 ± 27.81 | 50.75 | 0.77 | 62.79 ± 30.41 | 71.5 | 0.48 | 21.13 ± 21.12 | 10.25 | 0.99 | 48.69 ± 6.32 | 48.07 | 0.12 |
| | A3 | 31.51 ± 8.75 | 31.51 | 0.27 | 30.37 ± 30.13 | 14.59 | 0.97 | 28.42 ± 5.74 | 27.23 | 0.2 | 49.32 ± 6.82 | 49.44 | 0.13 |
| | A4 | 48.09 ± 1.05 | 48.09 | 0.02 | 53.74 ± 43.97 | 76.76 | 0.81 | 38.25 ± 8.93 | 39.69 | 0.23 | 49.16 ± 5.91 | 48.67 | 0.12 |
| | A5 | 40.22 ± 11.37 | 40.22 | 0.28 | 59.19 ± 43.12 | 81.87 | 0.72 | 56.52 ± 8.84 | 55.6 | 0.15 | 49.53 ± 6.18 | 49.01 | 0.12 |
| | A6 | 41.79 ± 6.97 | 41.79 | 0.16 | 17.54 ± 5.36 | 17.54 | 0.3 | 15.38 ± 15.08 | 7.2 | 0.98 | 46.22 ± 8.17 | 44.08 | 0.17 |

Lighting conditions (Brightness, Contrast for each case): A1= 1,-56; A2= 50,-25; A6= 90,-100; A4= Automatic scanner threshold (30,-69 White; 40,-69 Black); A3 and A5 values were selected for each background subtracting and adding 10 values of brightness from the automatic values, respectively, maintaining contrast values. Data was obtained from *A. thaliana* Col-0 genotype, infested with 50 *T. urticae* adults for 4 days; n= 3.

Table S4. Descriptive statistics for the estimated damaged areas identified on different backgrounds and lighting conditions. Values were obtained by analysing the genotypes: Bla-2, Col-0 and Kon.

| Genotype | Condition \ Program | | Assess | | | CompuEye | | | Ilastik | | | Photoshop | | |
|---|---|---|---|---|---|---|---|---|---|---|---|---|---|---|
| | | | Mean ± SD | Median | CV | Mean ± SD | Median | CV | Mean ± SD | Median | CV | Mean ± SD | Median | CV |
| Bla-2 | White background | W1 | 24.76 ± 15.82 | 24.76 | 0.64 | 14.58 ± 7.20 | 13.90 | 0.49 | 2.02 ± 1.13 | 1.96 | 0.56 | 14.11 ± 6.15 | 13.29 | 0.44 |
| | | W2 | 10.49 ± 3.68 | 10.49 | 0.35 | 12.89 ± 10.87 | 7.61 | 0.84 | 3.09 ± 1.62 | 3.00 | 0.52 | 14.87 ± 6.16 | 14.28 | 0.41 |
| | | W3 | 8.06 ± 2.65 | 8.06 | 0.33 | 14.59 ± 13.79 | 13.26 | 0.95 | 3.50 ± 1.24 | 3.60 | 0.35 | 15.35 ± 6.22 | 14.97 | 0.41 |
| | Black background | B1 | 3.50 ± 3.36 | 2.26 | 0.96 | 2.59 ± 2.57 | 1.16 | 0.99 | 2.64 ± 1.83 | 2.61 | 0.69 | 12.91 ± 6.38 | 12.49 | 0.49 |
| | | B2 | 4.30 ± 3.45 | 4.06 | 0.80 | 6.29 ± 5.68 | 5.30 | 0.90 | 3.63 ± 2.22 | 3.12 | 0.61 | 14.41 ± 6.50 | 13.77 | 0.45 |
| | | B3 | 5.67 ± 3.87 | 5.17 | 0.68 | - | - | - | 2.93 ± 1.82 | 3.18 | 0.62 | 14.58 ± 6.01 | 14.55 | 0.41 |
| Col-0 | White background | W1 | - | - | - | 10.21 ± 7.87 | 9.88 | 0.77 | 3.40 ± 1.73 | 4.06 | 0.51 | 22.29 ± 7.58 | 25.58 | 0.34 |
| | | W2 | - | - | - | 14.30 ± 11.97 | 12.21 | 0.84 | 4.21 ± 1.87 | 4.72 | 0.44 | 23.31 ± 7.41 | 26.35 | 0.32 |
| | | W3 | - | - | - | 14.80 ± 13.81 | 11.47 | 0.93 | 6.78 ± 2.07 | 7.10 | 0.31 | 23.74 ± 7.43 | 27.07 | 0.31 |
| | Black background | B1 | - | - | - | - | - | - | 5.58 ± 1.54 | 5.71 | 0.28 | 21.44 ± 7.36 | 24.30 | 0.34 |
| | | B2 | - | - | - | - | - | - | 6.25 ± 2.09 | 6.64 | 0.33 | 21.99 ± 7.56 | 24.95 | 0.34 |
| | | B3 | - | - | - | - | - | - | 7.56 ± 2.01 | 7.66 | 0.27 | 25.45 ± 8.90 | 28.26 | 0.35 |
| Kon | White background | W1 | 45.64 ± 26.44 | 40.00 | 0.58 | 40.26 ± 13.15 | 37.13 | 0.33 | 6.85 ± 2.95 | 6.55 | 0.43 | 60.19 ± 10.09 | 59.55 | 0.17 |
| | | W2 | 37.38 ± 16.63 | 37.19 | 0.44 | 45.66 ± 13.83 | 41.92 | 0.30 | 11.29 ± 3.88 | 12.30 | 0.34 | 62.14 ± 10.10 | 63.95 | 0.16 |
| | | W3 | 41.44 ± 17.91 | 40.08 | 0.43 | 49.96 ± 15.24 | 45.47 | 0.30 | 21.14 ± 10.56 | 20.24 | 0.50 | 59.66 ± 10.98 | 58.72 | 0.18 |
| | Black background | B1 | - | - | - | - | - | - | 3.60 ± 1.63 | 3.05 | 0.45 | 59.22 ± 9.60 | 59.72 | 0.16 |
| | | B2 | 38.82 ± 17.36 | 31.15 | 0.45 | 44.42 ± 18.24 | 36.49 | 0.41 | 19.49 ± 8.37 | 17.42 | 0.43 | 61.64 ± 9.99 | 62.93 | 0.16 |
| | | B3 | 61.39 ± 20.75 | 52.55 | 0.34 | 120.92 ± 37.24 | 113.23 | 0.31 | 33.75 ± 11.78 | 27.54 | 0.35 | 58.51 ± 10.39 | 57.00 | 0.18 |

Lighting conditions (Brightness, Contrast for each case): W2 and B2 = Automatic threshold (30,-20 White; 40,-10 Black); W1, W3, B1 and B3 values were selected for each background subtracting and adding 10 values of brightness, respectively, maintaining contrast values.
