# Supplementary material for: Saving time maintaining reliability: a new method for quantification of Tetranychus urticae damage in Arabidopsis whole rosettes
Source: BMC Plant Biol. 2020 Aug 27;20:397. doi: 10.1186/s12870-020-02584-0 (PMC7450957; doi:10.1186/s12870-020-02584-0)
Supplement: Supplementary file 2 — Additional file 2: Macro S1. Assess macro used to select area of interest and identify damaged areas. Macro S2. Fiji macro used to transform grey scale images to black/white. Macro S3. Fiji macro used to identify the pixel cluster size. Macro S4. Fiji macro used to calculate damaged areas on white/black images. Fig. S1. Frequency of appearance of damage clusters in Bla-2, Col-0 and Kon control rosettes. [file 12870_2020_2584_MOESM2_ESM.pdf]

Macro S1. Macro model used to select the area of interest and identify damaged areas on Assess 2.0. Sections underlined indicate variable planes/Colour spaces which depend on the analysed image. Pixel threshold values vary also depending on image, and are indicated by "###".

Batch_Process

Start_Batch_here

//Leaf

Select_leaf

<u>SetHSIColorSpace</u>

<u>Select_SaturationPlane</u>

SetLowThreshold(###)

SetHighThreshold(###)

ovl_Threshold_Image

Set_Leaf_AOI_ON

//Damage

Select_Lesion

<u>SetHSIColorSpace</u>

<u>Select_IntensityPlane</u>

SetLowThreshold(###)

SetHighThreshold(###)

ovl_Threshold_Image

//Rest of the steps

Select_Leaf

Ovl_Threshold_Image

Get_Leaf_Area

Select_Lesion

Ovl_Threshold_Image

Get_Lesion_Area

Set_SpreadSheet_ON

Get_Lesion_Area

Set_SpreadSheet_OFF

Macro S2. Commands used to transform grey scale images to black/white using ImageJ.

```
imageCount = nImages

n=nImages;

for(i=0,1; i<n; i++){

  setAutoThreshold("Default");

  run("Threshold...");

  setThreshold(0, 70);

  setOption("BlackBackground", false);

  run("Convert to Mask");

title=getTitle;


//Give the correct directory where you want to save the output images (marked red)

saveAs("TIFF", title);

close();

}
```

Macro S3. Commands that identified the pixel cluster size from which damage was assessed using ImageJ.

```
imageCount = nImages
n=nImages;
for(i=0,1; i<n; i++){
  setAutoThreshold("Default");
  run("Threshold...");
  setThreshold(129, 255);
  setOption("BlackBackground", false);
  run("Convert to Mask");
  run("Analyze Particles...", "size=0-Infinity show=Nothing display");
title=getTitle;

//Give the correct directory where you want to save the output images (marked red)
saveAs("TIFF", title);
close();
}
```

Macro S4. Commands used to calculate damaged areas on white/black images using ImageJ.

```
imageCount = nImages

n=nImages;

for(i=0,1; i<n; i++){

  setAutoThreshold("Default");

  run("Threshold...");

  setThreshold(129, 255);

  setOption("BlackBackground", false);

  run("Convert to Mask");

  run("Analyze Particles...", "size=37-Infinity show=Nothing clear include summarize");

title=getTitle;


//Give the correct directory where you want to save the output images (marked red)

saveAs("TIFF", title);

close();

}
```

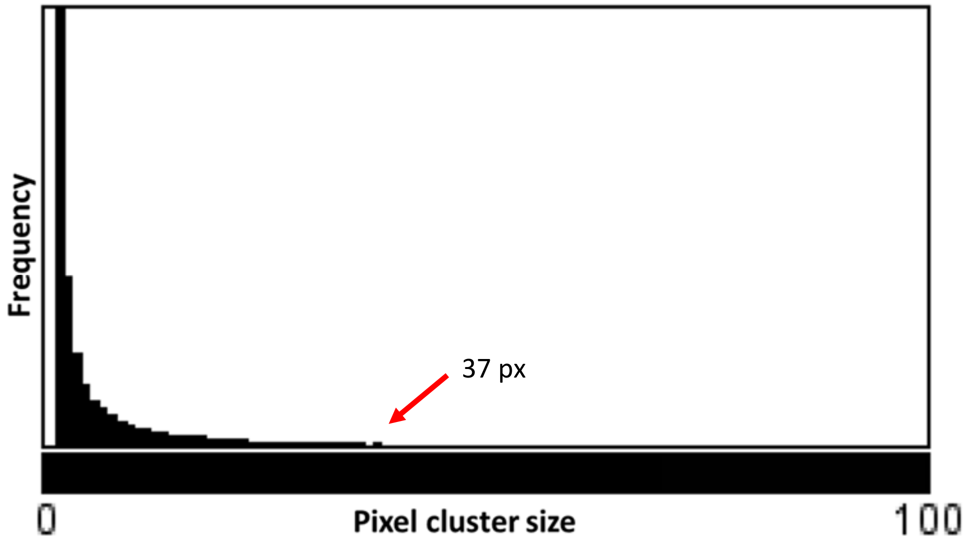

Fig. S1 Frequency of appearance of damage clusters on Bla-2, Col-0 and Kon control rosettes. A size threshold to discriminate background noise is selected using the present frequency graph. The biggest cluster with the highest frequency (37) is selected as the cutting point from which damage is considered.
