# Supplementary material for: Saving time maintaining reliability: a new method for quantification of Tetranychus urticae damage in Arabidopsis whole rosettes
Source: BMC Plant Biol. 2020 Aug 27;20:397. doi: 10.1186/s12870-020-02584-0 (PMC7450957; doi:10.1186/s12870-020-02584-0)
Supplement: Supplementary file 3 — Additional file 3: Table S1. Statistical R output for GLM analysis. The test compares the damaged areas identified by the automatic methods under six lighting conditions and white/black backgrounds for the Col-0 genotype. Table S2. Statistical R output for GLM analysis. The test compares the damaged areas identified by the automatic methods under three lighting conditions and white/black backgrounds for the genotypes: Bla-2, Col-0 and Kon. Table S3. Pair-wise comparison with Bonferroni correction comparing method results under different lighting conditions. The test compares the damaged areas identified by the automatic methods under three lighting conditions on white/black backgrounds for the genotypes: Bla-2, Col-0 and Kon. Table S4. Statistical R output for GLM analysis. The test compares the damaged areas identified by the automatic methods under three lighting conditions and white/black backgrounds for the controls of the genotypes: Bla-2, Col-0 and Kon. Table S5. Pair-wise comparison with Bonferroni correction comparing control rosette results under different lighting conditions. The test compares the damaged areas identified by the automatic methods under three lighting conditions on white/black backgrounds for the control of the genotypes, Bla-2, Col-0 and Kon. [file 12870_2020_2584_MOESM3_ESM.pdf]

Table S1. Statistical R output for GLM analysis. The test compares the damaged areas identified by the automatic methods under six lighting conditions and white/black backgrounds for the Col-0 genotype.

	Df	Deviance	Resid. Df	Resid. Dev	Pr(>Chi)	Chi sqrt
NULL			98	91.377		
Program	3	7.9474	95	83.429	< 0.001 ***	31,34
Background	1	10.1465	94	73.283	< 0.001 ***	40,01
ConditionID	6	20.0818	88	53.201	< 0.001 ***	79,19
Program:Background	2	7.6047	86	45.596	< 0.001 ***	29,99
Program:ConditionID	16	16.1922	70	29.404	< 0.001 ***	63,85
Background:ConditionID	4	1.3035	66	28.101	0.2732	5,14
Program:Background:ConditionID	4	1.8977	62	26.203	0.1124	7,484

Signif. codes: 0 ‘***’ 0.001 ‘**’ 0.01 ‘*’ 0.05 ‘.’ 0.1 ‘ ’ 1

The factors under analysis were: Program (the three automatic methods and the standard); Background (white/black); ConditionID (an array of six lighting conditions).

Table S2. Pair-wise comparison with Bonferroni correction comparing method results under different lighting conditions. The test compares the damaged areas identified by the automatic methods under three lighting conditions on white/black backgrounds for the genotypes: Bla-2, Col-0 and Kon.

Program	ConditionID	lsmean	SE	df	asymp.LCL	asymp.UCL	.group
Assess	W1	0.0322	0.00699	Inf	0.0107	0.0538	a
Photoshop	W3	0.0413	0.00432	Inf	0.0280	0.0546	a
Photoshop	B3	0.0417	0.00441	Inf	0.0281	0.0552	a
Photoshop	W2	0.0421	0.00443	Inf	0.0284	0.0557	a
Photoshop	B2	0.0437	0.00461	Inf	0.0295	0.0579	a
Photoshop	W1	0.0441	0.00466	Inf	0.0298	0.0585	a
Photoshop	B1	0.0470	0.00501	Inf	0.0316	0.0624	a
CompuEye	W3	0.0520	0.00750	Inf	0.0290	0.0751	a
CompuEye	W2	0.0565	0.00829	Inf	0.0310	0.0820	a
Assess	W2	0.0583	0.01329	Inf	0.0174	0.0992	a
Assess	W3	0.0637	0.01512	Inf	0.0172	0.1103	a
CompuEye	W1	0.0638	0.01028	Inf	0.0321	0.0954	a
Ilastik	W3	0.1602	0.01866	Inf	0.1027	0.2176	b
Ilastik	B2	0.1622	0.01846	Inf	0.1053	0.2190	bc
Ilastik	B3	0.1679	0.02124	Inf	0.1026	0.2333	bc
Ilastik	W2	0.2167	0.02339	Inf	0.1447	0.2887	bc
Ilastik	B1	0.2785	0.02978	Inf	0.1868	0.3702	bc
Ilastik	W1	0.3118	0.03592	Inf	0.2012	0.4223	c
Assess	B1	nonEst	NA	NA	NA	NA	
CompuEye	B1	nonEst	NA	NA	NA	NA	
Assess	B2	nonEst	NA	NA	NA	NA	
CompuEye	B2	nonEst	NA	NA	NA	NA	
Assess	B3	nonEst	NA	NA	NA	NA	
CompuEye	B3	nonEst	NA	NA	NA	NA	

Lighting conditions (Brightness, Contrast for each case): W2 and B2 = Automatic threshold (30,-20 White; 40,-10 Black); W1, W3, B1 and B3 values were selected for each background subtracting and adding 10 values of brightness, respectively, maintaining contrast values.

Table S3. Statistical R output for GLM analysis. The test compares the damaged areas identified by the automatic methods under three lighting conditions and white/black backgrounds for the genotypes: Bla-2, Col-0 and Kon.

```
                             Df Deviance Resid. Df Resid. Dev  Pr(>Chi)        Chi sqrt
NULL                                           412     481.65
Program                       3  142.662       409     338.99 < 0.001 ***     76,01
ConditionID                   5   21.797       404     317.19 < 0.001 ***     21,8
Genotype                      2  141.420       402     175.77 < 0.001 ***     72,11
Program:ConditionID          15   42.953       387     132.82 < 0.001 ***     42,95
Program:Genotype              6   29.225       381     103.59 < 0.001 ***     29,22
ConditionID:Genotype         10    1.430       371     102.16 0.9991366       1,43
Program:ConditionID:Genotype 22    9.280       349      92.88 0.9917398       9,28
---
Signif. codes:  0 ‘***’ 0.001 ‘**’ 0.01 ‘*’ 0.05 ‘.’ 0.1 ‘ ’ 1
```

The factors under analysis were: Program (the three automatic methods and the standard); Background (white/black, nested inside the factor ConditionID); ConditionID (three lighting conditions); Genotype (Bla-2, Col-0, Kon).

Table S4. Statistical R output for GLM analysis. The test compares the damaged areas identified by the automatic methods under three lighting conditions and white/black backgrounds for the controls of the genotypes: Bla-2, Col-0 and Kon.

	Df	Deviance	Resid. Df	Resid. Dev	Pr(>Chi)		Chi sqrt
NULL			431	566.16			
Program	2	320.21	429	245.96	< 0.001	***	72,11
ConditionID	5	34.01	424	211.95	< 0.001	***	82,78
Genotype	2	1.05	422	210.90	< 0.001	***	14,61
Program:ConditionID	10	45.60	412	165.30	< 0.001	***	96,97
Program:Genotype	4	30.58	408	134.72	< 0.001	***	79,52
ConditionID:Genotype	10	79.56	398	55.16	< 0.001	***	96,97
Program:ConditionID:Genotype	20	25.78	378	29.38	< 0.001	***	120,6

Signif. codes: 0 ‘***’ 0.001 ‘**’ 0.01 ‘*’ 0.05 ‘.’ 0.1 ‘ ’ 1

The factors under analysis were: Program (the three automatic methods); Background (white/black, nested inside the factor ConditionID); ConditionID (three lighting conditions); Genotype (Bla-2, Col-0, Kon).

Table S5. Pair-wise comparison with Bonferroni correction comparing control rosette results under different lighting conditions. The test compares the damaged areas identified by the automatic methods under three lighting conditions on white/black backgrounds for the control of the genotypes: Bla-2, Col-0 and Kon.

Program	ConditionID	Genotype	lsmean	SE	df	asymp.LCL	asymp.UCL	.group
CompuEye	A4	Kon	0.0198	0.00188	Inf	0.0136	0.0260	a
Assess	A4	Kon	0.0247	0.00233	Inf	0.0169	0.0324	ab
CompuEye	A6	Bla	0.0273	0.00258	Inf	0.0187	0.0358	abc
Assess	A1	Bla	0.0284	0.00268	Inf	0.0195	0.0373	abc
Assess	A2	Bla	0.0356	0.00337	Inf	0.0244	0.0467	abcd
CompuEye	A3	Bla	0.0357	0.00338	Inf	0.0245	0.0469	abcd
Assess	A3	Bla	0.0357	0.00338	Inf	0.0245	0.0469	abcd
CompuEye	A2	Bla	0.0403	0.00381	Inf	0.0276	0.0529	bcde
Assess	A3	Col	0.0409	0.00387	Inf	0.0281	0.0537	bcde
CompuEye	A2	Col	0.0411	0.00389	Inf	0.0282	0.0540	bcde
CompuEye	A1	Bla	0.0416	0.00394	Inf	0.0286	0.0547	bcde
CompuEye	A1	Col	0.0428	0.00405	Inf	0.0294	0.0563	bcdef
Assess	A1	Col	0.0436	0.00412	Inf	0.0299	0.0572	bcdef
Assess	A2	Col	0.0440	0.00416	Inf	0.0302	0.0578	bcdef
CompuEye	A3	Col	0.0471	0.00446	Inf	0.0323	0.0618	cdefg
CompuEye	A6	Col	0.0487	0.00460	Inf	0.0334	0.0639	cdefg
CompuEye	A3	Kon	0.0520	0.00492	Inf	0.0357	0.0682	defgh
CompuEye	A1	Kon	0.0520	0.00492	Inf	0.0357	0.0683	defgh
CompuEye	A2	Kon	0.0521	0.00493	Inf	0.0358	0.0685	defgh
Assess	A3	Kon	0.0642	0.00608	Inf	0.0441	0.0844	defghi
Assess	A2	Kon	0.0686	0.00649	Inf	0.0471	0.0900	efghi
Assess	A1	Kon	0.0695	0.00658	Inf	0.0477	0.0913	efghi
CompuEye	A5	Bla	0.0755	0.00714	Inf	0.0518	0.0991	fghij
Assess	A6	Col	0.0837	0.00792	Inf	0.0575	0.1100	ghijk
Assess	A4	Col	0.0886	0.00838	Inf	0.0608	0.1163	hijk
CompuEye	A5	Kon	0.0971	0.00919	Inf	0.0666	0.1275	ijk
Ilastik	A3	Kon	0.1000	0.00946	Inf	0.0687	0.1313	ijkl
Assess	A5	Col	0.1157	0.01095	Inf	0.0794	0.1519	ijkl
Assess	A5	Kon	0.1272	0.01204	Inf	0.0873	0.1671	jklm
CompuEye	A5	Col	0.1285	0.01216	Inf	0.0882	0.1687	jklm
Assess	A6	Bla	0.1396	0.01321	Inf	0.0958	0.1833	klmn
CompuEye	A4	Col	0.1397	0.01322	Inf	0.0959	0.1834	klmn
Assess	A5	Bla	0.1798	0.01702	Inf	0.1235	0.2362	lmno
Assess	A4	Bla	0.1808	0.01710	Inf	0.1241	0.2374	lmno
CompuEye	A6	Kon	0.2147	0.02032	Inf	0.1474	0.2820	mno
Ilastik	A4	Kon	0.2162	0.02046	Inf	0.1485	0.2840	mnop
CompuEye	A4	Bla	0.2389	0.02261	Inf	0.1641	0.3138	nop
Ilastik	A2	Kon	0.3068	0.02903	Inf	0.2107	0.4030	opq
Ilastik	A5	Kon	0.3911	0.03701	Inf	0.2685	0.5137	pqr
Ilastik	A4	Col	0.5070	0.04797	Inf	0.3481	0.6658	qrs
Ilastik	A1	Kon	0.6379	0.06036	Inf	0.4379	0.8378	rst
Ilastik	A6	Col	0.6421	0.06076	Inf	0.4408	0.8433	rst
Ilastik	A1	Bla	0.6593	0.06239	Inf	0.4527	0.8659	rst
Ilastik	A5	Col	0.6772	0.06408	Inf	0.4650	0.8895	rst
Ilastik	A3	Bla	0.6815	0.06449	Inf	0.4679	0.8951	rst
Ilastik	A4	Bla	0.6837	0.06469	Inf	0.4694	0.8979	rst
Ilastik	A3	Col	0.7152	0.06768	Inf	0.4911	0.9394	stu
Ilastik	A2	Col	0.7906	0.07481	Inf	0.5428	1.0383	stu
Ilastik	A1	Col	0.8968	0.08486	Inf	0.6157	1.1778	stu
Ilastik	A5	Bla	0.9541	0.09028	Inf	0.6550	1.2531	tu
Ilastik	A2	Bla	1.0349	0.09793	Inf	0.7106	1.3593	tu
Ilastik	A6	Bla	1.0803	0.10222	Inf	0.7417	1.4189	tu
Assess	A6	Kon	1.2485	0.11814	Inf	0.8572	1.6398	u
Ilastik	A6	Kon	2.5566	0.24193	Inf	1.7553	3.3579	v
