# Supplementary material for: Saving time maintaining reliability: a new method for quantification of Tetranychus urticae damage in Arabidopsis whole rosettes
Source: BMC Plant Biol. 2020 Aug 27;20:397. doi: 10.1186/s12870-020-02584-0 (PMC7450957; doi:10.1186/s12870-020-02584-0)
Supplement: Supplementary file 4 — Additional file 4. Flow chart depicting the general steps for the three automatic analysis tested. [file 12870_2020_2584_MOESM4_ESM.pptx]

## Slide 1
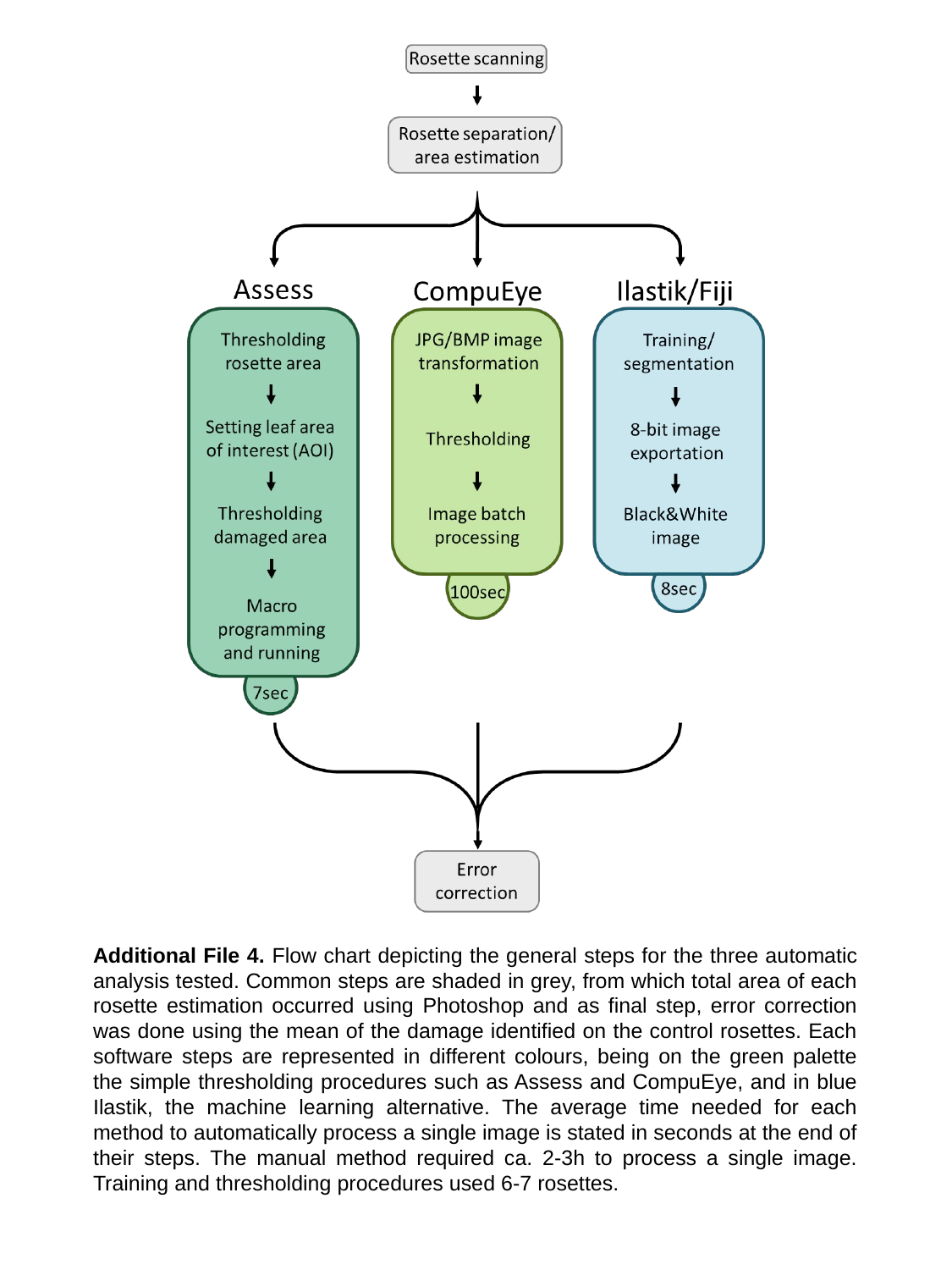

Additional File 4. Flow chart depicting the general steps for the three automatic analysis tested. Common steps are shaded in grey, from which total area of each rosette estimation occurred using Photoshop and as final step, error correction was done using the mean of the damage identified on the control rosettes. Each software steps are represented in different colours, being on the green palette the simple thresholding procedures such as Assess and CompuEye, and in blue Ilastik, the machine learning alternative. The average time needed for each method to automatically process a single image is stated in seconds at the end of their steps. The manual method required ca. 2-3h to process a single image. Training and thresholding procedures used 6-7 rosettes.
