# Supplementary material for: Saving time maintaining reliability: a new method for quantification of Tetranychus urticae damage in Arabidopsis whole rosettes
Source: BMC Plant Biol. 2020 Aug 27;20:397. doi: 10.1186/s12870-020-02584-0 (PMC7450957; doi:10.1186/s12870-020-02584-0)
Supplement: Supplementary file 5 — Additional file 5. Detailed protocol for the spider mite damage quantification in Arabidopsis whole rosettes. [file 12870_2020_2584_MOESM5_ESM.docx]

**PHOTOSHOP**

1. Open the image containing all the rosettes and individualise them by cropping, saving them in the same format.
2. Whole rosette area calculation: On the original image containing all the rosettes.
   1. Using rectangular selection, select a rosette.
   2. Choose Select/Colour range
   3. Using the Eyedropper tool in the dialog box, select the colours that allows the identification of the rosette from the background. Select OK
   4. Go to the histogram, update the information and copy the number of pixels.

**ILASTIK**

1. Open Ilastik, select “Pixel classification” and save the project in the desired folder.
2. Introduce the images that will be used for training in “Input data”.
3. Select, on “Feature selection”, the three options under the one-pixel option.
4. Create three labels for damage, leaf and background.
5. Select the “Leaf” label and, using the brush in 1-pixel size, paint the center of the rosette and some of the leaf petiole.
6. Select the “Background” label and paint the area surrounding the rosette. If the area has some noise, like threads or other colored materials, include them below the “Background” label so the program identifies and labels it as background and not rosette.
7. Click on “Live update” and then uncheck “Probability” and check “Segmentation”, from the image formed, try to cover all the background identified as leaf with the pencil for background. Correctly identify background from leaf specially in the borders of the leaves. Unchecking the “Leaf Segmentation” layer (clicking on the eye), the discrimination is easier done.
8. Select the “Damage” label and identify the damage in the leaves, verify the selection done in the other damaged replicates. All the damage should be selected, no matter if non damaged areas are selected, they will be corrected using the controls.
9. Reduce the amount of false damage using the control rosettes, paying special attention to the petioles and the trichomes. To eliminate further non damaged area, uncheck the Damage layer, and with the Leaf and Background layers on, mark the darker pixels using the pencil on Leaf mode.
10. Import all the images that will be analyzed on the “Batch prediction input selections” tab.
11. On the “Batch prediction output location” tab, select “Simple segmentation” for the “Export Source” foldable list.
12. Select for the “Export settings”:
    1. Convert to data type: Signed 8-bit
    2. Format: jpeg
    3. File: Select the file output location for the segmented images.

NOTE: The training data (six to seven images) are not in the test set. Training will be necessary for each genotype/lighting condition. Therefore, each of them will have their own training file (.ilp).

**FIJI**

1. Open Fiji, select Plugins/New/Macro.
2. In the new Window, click on File/Open
3. Surf and open the three macros used to transform grayscale images to black/white, identify the noise and calculate the damaged area (Additional file 3, Macros S2-S4). They will be opened in the same window as tabs.
4. Drag the segmented images exported from Ilastik to Fiji, be sure there is no scale by checking in Analyze/Set scale
5. In the extra window of Fiji, select the tab with the macro that transforms images from grayscale to black/white (Additional file 3, Macro S2), and click the button Run.
6. If no address was given to the program to save the images, they will be in the program directory folder (E.g.: D:\programs\Image J Fiji\Fiji.app).
7. Drag the images processed by Fiji into Fiji, open the macro that identifies background noise (Additional file 3, Macro S3), and click the button Run.
8. A window called Results will pop up, inside that window, click Results/Distribution. In the new window called Distribution, set Parameter: Area; Uncheck “Automatic binning” and specify 100 bins with a range from 0-100.
9. Maximize the area distribution graphic to see the details. Select the size exclusion criteria by using the cluster with the lowest frequency of appearance. By locating the mouse over the bars of the frequency graph, the information of the size of the cluster can be annotated. Put the cursor over the last bar on the tail of the distribution and get the size of the cluster it represents. (An optimal cleaning process renders a short tail, around 37 pixels)
10. Close all the windows but the Fiji and Macro windows.
11. Drag the images obtained from step 6 into Fiji.
12. Open the macro used to calculate damaged area (Additional file 3, Macro S4), in the body of the programming code, use the value selected in the previous step inside the line that reads: "size=PutYourSizeHere-Infinity show=Nothing clear include summarize" and click the button Run.
13. Copy the pixel information given in the newly opened window.
14. To transform the pixel information to cm^2^, a process of calibration (scaling), will be required in order to know the number of pixels in a given area of an image. After the scaling procedure, the pixels can be transformed following the equation:

$$A=\frac{P_{r}}{P_{c}}*A_{c}$$

Where A accounts for the rosette damaged area; $P_{r}$ represents pixels of the rosette of interest, $P_{c}$ the pixels inside the area used for calibration and $A_{c}$ the area for calibration.

The previous steps will be necessary to process the images the first time. For daily use of the protocol, once the training file of Ilastik is obtained, the procedure will use the steps from Photoshop, the steps 10-12 from Ilastik and all the steps from Fiji, excluding 7-10.
